# X-ray imageable, drug-loaded hydrogel that forms at body temperature for image-guided, needle-based locoregional drug delivery

**DOI:** 10.1038/s41598-024-64189-z

**Published:** 2024-06-10

**Authors:** Jose F. Delgado, William F. Pritchard, Nicole Varble, Tania L. Lopez-Silva, Antonio Arrichiello, Andrew S. Mikhail, Robert Morhard, Trisha Ray, Michal M. Havakuk, Alex Nguyen, Tabea Borde, Joshua W. Owen, Joel P. Schneider, John W. Karanian, Bradford J. Wood

**Affiliations:** 1 National Institutes of Health, Center for Interventional Oncology, Radiology and Imaging Sciences, Clinical Center, Bethesda, MD USA; 2 Fischell Department of Bioengineering, University of Maryland, College Park, MD USA; 3 Philips Healthcare, Cambridge, MA USA; 4 Chemical Biology Laboratory, National Cancer Institute, National Institutes of Health, Center for Cancer Research, Frederick, MD USA; 5 UOS of Interventional Radiology, Department of Diagnostic and Interventional Radiology, Ospedale Maggiore di Lodi, Largo Donatori del Sangue, Lodi, Italy; 6 Interventional Radiology Department, Tel-Aviv Sourasky Medical Center, Tel-Aviv, Israel; 7 Computer Science Department, Stanford University, Stanford, CA USA

**Keywords:** Cancer, Medical research, Oncology, Engineering

## Abstract

Liver cancer ranks as the fifth leading cause of cancer-related death globally. Direct intratumoral injections of anti-cancer therapeutics may improve therapeutic efficacy and mitigate adverse effects compared to intravenous injections. Some challenges of intratumoral injections are that the liquid drug formulation may not remain localized and have unpredictable volumetric distribution. Thus, drug delivery varies widely, highly-dependent upon technique. An X-ray imageable poloxamer 407 (POL)-based drug delivery gel was developed and characterized, enabling real-time feedback. Utilizing three needle devices, POL or a control iodinated contrast solution were injected into an ex vivo bovine liver. The 3D distribution was assessed with cone beam computed tomography (CBCT). The 3D distribution of POL gels demonstrated localized spherical morphologies regardless of the injection rate. In addition, the gel 3D conformal distribution could be intentionally altered, depending on the injection technique. When doxorubicin (DOX) was loaded into the POL and injected, DOX distribution on optical imaging matched iodine distribution on CBCT suggesting spatial alignment of DOX and iodine localization in tissue. The controllability and localized deposition of this formulation may ultimately reduce the dependence on operator technique, reduce systemic side effects, and facilitate reproducibility across treatments, through more predictable standardized delivery.

## Introduction

Localized needle-based percutaneous delivery of anti-cancer agents directly into tumors offers significant benefits over traditional intravenous drug delivery^1^. This method potentially improves therapeutic outcomes and bioavailability by enabling higher local drug concentrations^2,3^. Intratumoral injections have been increasing in popularity^2^. Anti-cancer agents that may be delivered include immunotherapy options^4–8^ and chemotherapy drugs^9–11^.

To improve drug delivery kinetics and localized retention, drugs can be incorporated into hydrogels for delivery. This approach reduces the leakage of injected drugs towards healthy tissue by increasing formulation viscosity^12–18^. Potential benefits of hydrogels include sustained release of cargo and high concentration of doses at the target tissue over longer periods. Hydrogels can be delivered via minimally invasive needles, with potential for enhanced reproducibility through reduction in technical variation, leading to more predictable drug distribution. Loading anti-cancer agents into hydrogels has been reported extensively^19^ with an existing variety of mechanisms triggering gelation that include thermosensitive, pH-sensitive, photosensitive, or dual-sensitive^20–23^ stimuli.

An example of a thermosensitive hydrogel is Poloxamer 407 (POL), a triblock copolymer that self-aggregates into micelles due to its amphiphilic nature^24^. POL is used as an inactive ingredient approved in drug formulations for intratympanic, oral, and topical applications^25^. The formation of a 3D-ordered structure is driven by the critical micellization concentration and temperature^24,26^. The addition of contrast agents enables visualization in image-guided interventions.

POL as a liquid embolic and iodine contrast agent have been used previously in preclinical studies^12,27^. The combination was imageable under X-rays allowing image-guided catheter and needle-based delivery of hydrogels. Another formulation containing iodine such as a polypeptide-based hydrogel was used for intratumoral injections^12^.

Despite the precision in needle placement using imaging techniques, injection techniques vary and lacks standardization of methodologies, such as injection parameters of infusion rate, volume, and needle type^28^. Injection parameters to optimize volumetric distribution of hydrogels have been tested in an ex vivo bovine liver model^29^ with multiple needle types, however, further systematic characterization of hydrogels and optimization of injection parameters are needed to better predict performance to facilitate translation to clinic.

To fill this gap, this study aimed to demonstrate that POL formulations, known for their sustained drug release properties, are effective for localized injections irrespective of the variations in needle devices and injection parameters. This work was intended to contribute towards standardization of intratumoral injection methods for further study in clinical settings.

## Results

### Development and properties of an X-ray imageable drug-loaded POL formulation

**Figure 1:**
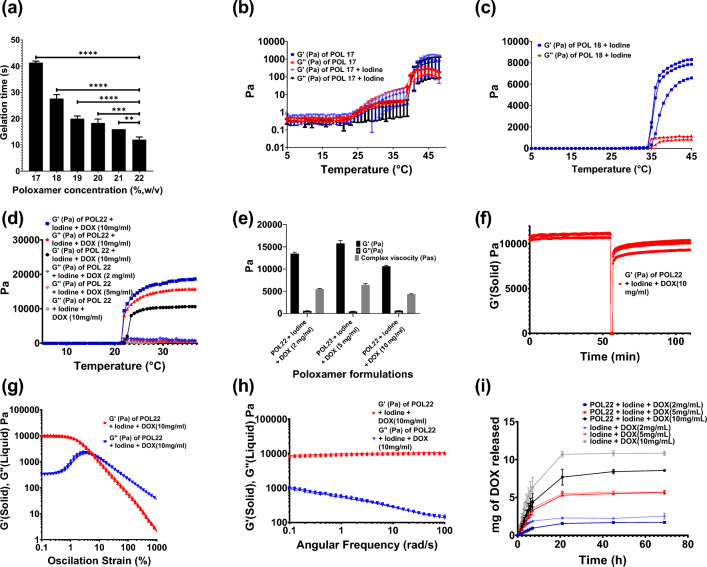
Gelation time, viscoelastic properties, and drug elution profiles. (**a**) Gelation times of various POL concentrations ranging from 17 to 22% (w/v) (n = 3). (**b**) Temperature ramp from 5 to 37 °C for imageable (n = 6) and non-imageable POL17 (n = 6), and (**c**) imageable POL18 (n = 3). (**d**) Temperature ramp from 5 to 37 °C for imageable POL22 and 2, 5, and 10 mg/ml of DOX in normal saline along with their respective G′ and G′′ (n = 3 for all DOX concentrations). (**e**) Storage modulus (G′), loss of modulus (G′′), and complex viscosity at 37 °C for imageable POL with 2, 5, and 10 mg/mL of DOX (n = 3 for all DOX concentrations). (**f**) Thixotropic behavior of POL22 containing 10 mg/mL of DOX (n = 3). (**g**) Oscillation strain sweep for iodinated POL22 formulations containing with 10 mg/mL of DOX (n = 3). (**h**) Frequency sweep for iodinated POL22 formulations with 10 mg/mL of DOX (n = 3). (**i**) Drug elution profiles for 2, 5, and 10 mg/mL DOX from the gel are shown along with solutions of DOX and iodine as control groups (n = 3 for each formulation). Error bars represent standard deviations of mean value. **p < 0.01, ***p < 0.001, and ****p < 0.0001, from one-way ANOVA statistical test.

To optimize POL formulation to be used as an injectable material, six different concentrations of POL (17, 18, 19, 20, 21, and 22%, w/v) were characterized for gelation times and then three different concentrations of POL (17, 18, and 22%, w/v) were systematically characterized for rheology. The gelation times decreased as the concentration of POL increased (Fig. 1a). The gelation temperature also decreased with increasing POL concentration and decreased after adding the iodinated contrast agent iodixanol to the formulations (Fig. 1b and c; Fig. S1). POL22 was selected as a suitable concentration for successful localized injection, balancing injection time and gel formation once at body temperature because of its rapid gelation time of at body temperature (37 °C) and gelation temperature below 37 °C. The gelation time for POL22 was 12 ± 1 s (Fig. 1a). The gelation temperatures for POL22 + iodine containing 2, 5, and 10 mg/mL of DOX at 37 °C were 22 ± 0.2 °C, 21.9 ± 0.2 °C, and 23 ± 0.3 °C, respectively. Overall, adding drug to the system did not affect the gelation temperature (Fig. 1d).

POL22, with iodixanol and DOX, showed viscoelastic properties characteristic for gels as evidenced by G′ exceeding G′′ values (Fig. 1d) at 37 °C. For POL22 containing iodine and 10 mg/mL of DOX, the G′ was 10,637 ± 214.8 Pa, G′′ was 618.12 ± 56.9 P, and complex viscosity of 4350 ± 88 Pas. The increase of DOX from 2 to 5 mg/mL resulted in a 1.2-fold increase in G’ (p = 0.0083) and complex viscosity (p = 0.0021), whereas an increase from 5 to 10 mg/mL resulted in a 0.7-fold decrease in these values (p < 0.0001 for G′, and p = 0.0019 for complex viscosity) (Fig. 1e).

Although we envision injecting these materials as liquids for in situ gelation, they can be delivered by needle injection as gels. POL22 with iodixanol and DOX formulations could recover their initial G′ values (solid-like behaviour) after high strain. The addition of iodixanol or DOX does not affect the ability to recover G′ of the gels, therefore, the formulations are thixotropic (Fig. 1f). When 10 mg/mL of DOX were incorporated into POL22 + iodixanol, the flow point value recorded was 8.2% ± 1 (Fig. 1g). The frequency range tested showed gel behavior at 37 °C for POL22 with iodine and DOX as G′ exceeded G′′, indicating that the material behaved as a viscoelastic gel and remained stable over time (Fig. 1h).

**Figure 2:**
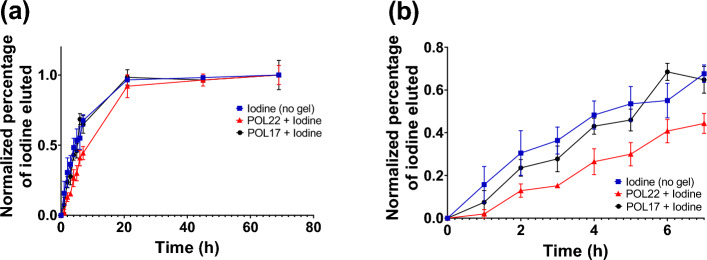
In vitro release of iodine. (**a**) Elution profiles for in vitro release of iodine from POL17 and POL22 gels and control, iodine without gel. (**b**) Detailed view of elution profiles of (**a**) from 0 to 7 h. Error bars represent standard deviations of mean value. n = 3 for each experiment. Statistical analysis performed with one-way ANOVA.

In vitro release profiles were evaluated on iodine and drug formulations with and without gel since these elution profiles could be helpful for predicting the imageability of the x-ray contrast agent in vivo. The in vitro release profile of DOX from the iodinated POL22 formulation was evaluated and compared to control groups (Figs. 1i and S6). Over the initial 7 h, the formulations with and without gel exhibited a zero-order kinetic release. The release rate was estimated to be 0.3 ± 0.0, 0.5 ± 0.1, and 0.9 ± 0.1 mg/h for 2, 5, and 10 mg of DOX respectively in the control groups (Iodine + DOX; no gel). For the iodinated POL22 formulation, the release rates of the drug were 0.1 ± 0.0, 0.5 ± 0.0, and 0.6 ± 0.2 mg/h for gel containing 2, 5, and 10 mg/mL of DOX respectively. With the material contained within a dialysis cassette, POL extended the the time for 50% of iodine release from the dialysis cassette compared to free iodine in normal saline solutions (control) (Fig. 2a and b). Fifty percent of the iodine for the control experiment eluted at 4.8 h ± 1.3. In contrast, the half-lives of the iodine released from POL17 and POL22 were 4.8  ± 0.7 h and 8.6  ± 1.5 h respectively. POL22 extended the time for 50% iodine release by 1.8 times compared to the control experiment (p = 0.02, n = 3).

**Figure 3:**
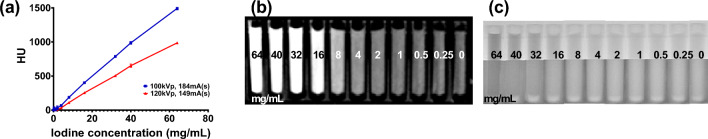
Imaging of iodinated contrast. (**a**) Attenuation in Hounsfield units (HU) of iodixanol as measured on CBCT versus iodine concentration using 100 and 120 kVp imaging protocols (n = 3). (**b**) Multiplanar reformatted CBCT image. (**c**) Fluoroscopic image of iodine dilutions. Iodine concentration, mg I/mL, are shown for each tube.

The in vitro imageability of iodine dilutions with CBCT and fluoroscopy was determined qualitatively and quantitatively with different imaging protocols. The relationship between Hounsfield units (HU), a measure of radiodensity on CT images, and iodine concentration (mg/mL) demonstrated linearity across the 120 and 100 kVp protocols, with correlation r^2^ values of 1. Correspondingly, the slope of these linear plots was 15.7 and 23.9 (Fig. 3a). An illustrative CBCT image acquired at 100 kVp demonstrating iodine serial dilutions is provided. The qualitative visibility was observed with an iodine concentration as low as 4–8 mg/mL (Fig. 3b). The iodine formulations surpassing the Rose criterion (CNR > 2.5) stood at 2 mg/mL for both imaging protocols at 120 kVp and 100 kVp. The value represents the lower limit of iodine detection in this idealized phantom. Lower kVp provides higher signal-to-noise ratio in X-ray images^30^ compared to 120 kVp and higher sensitivity. Radiographic and fluoroscopic imaging are less sensitive to iodine than CT, but the density difference at the higher concentrations remains evident (Fig. 3b and c).

### Ex vivo evaluation of injection parameters for three devices in bovine liver

Three needle types were studied: a single end-hole needle (SEHN), a multiple side hole needle (MSHN), and multiple prong injection needle (MPIN). To evaluate the maximum volume that can be localized before extravasation, this critical volume was evaluated ex vivo, using injection volumes equivalent to that of 2, 3, and 4 cm diameter spheres, 4 mL, 8.6 mL, and 14 mL, respectively. The critical volume to perform the ex vivo evaluation of injection parameters with iodinated-POL was 4 mL, as 8.6 mL and 14 mL volumes led to material leakage in the majority of the tested times. In some cases, 8.6 mL, and 14 mL injected gels were localized (Fig. S7).

**Figure 4:**
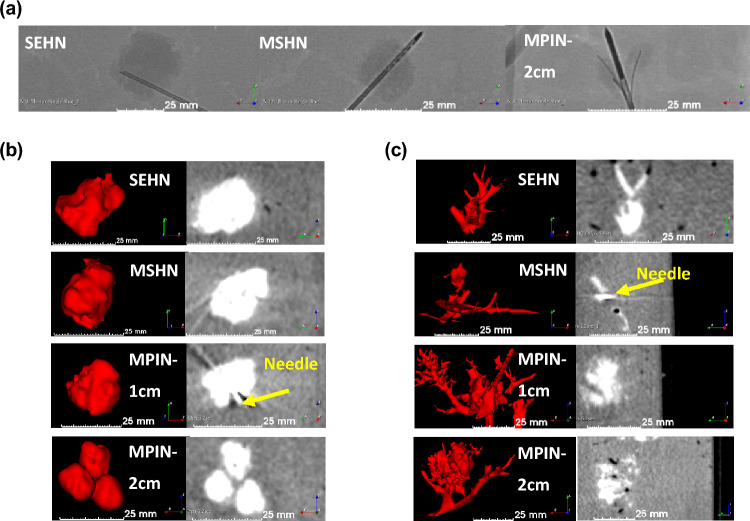
Fluoroscopic and cone beam CT (CBCT) imaging following injection of 4 mL of POL22 with iodine or iodine alone at 10 mL/h into ex vivo bovine liver. Three needles were used: SEHN, MSHN, and MPIN deployed at 1 cm (MPIN-1cm) and deployed at 2 cm (MPIN-2cm). (**a**) Fluoroscopic images following injection of POL22 with iodine with SEHN, MSHN and MPIN-2cm showing the gel deposition (higher density areas) relative to the needle locations. (**b**) POL22 with iodine injections. 3D surface renderings (left) and corresponding cross-sectional images (right) based on CBCT imaging. (**c**) Injections of iodine alone. 3D surface renderings (left) and corresponding cross-sectional images (right) based on CBCT imaging.

Radiological and fluoroscopic Imageability of the gels were also assessed. POL22 + Iodine gels were imageable under fluoroscopic single image acquisitions during and after injection in bovine livers. Four milliliters of gel were injected with SEHN 18G, MSHN 19G, and MPIN 18G. Rounded morphologies were obtained for gels injected with SEHN (Fig. 4a, left) and MSHN (Fig. 4a, middle) The MPIN with the needles deployed 2 cm (MPIN-2cm) (Fig. 4a, right), produced three localized gel depots. The fluoroscopic images demonstrated localized injections with POL22 gels in tissue.

The morphological and size characterization of gels post-injection was determined for varying injection parameters. Three-dimensional volumetric reconstructions were generated following segmentation of 4 mL injections of POL22 + Iodine into ex vivo bovine livers, imaged with CBCT at 80 kVp (Fig. 4b). The injections were performed at 10, 100, and 1000 mL/h using SEHN 18G and MSHN 19G. Needle sizes of 18G and 19G were used because of their capability to deliver viscous materials such as POL. For the case of MPIN 18G needles, 10 and 100 mL/h were used. The gels formed spheroidal and ellipsoidal shapes post-injection. SEHN 18G and MSHN 19G produced depositions measuring 21.3 ± 0.7 mm × 20.7 ± 0.6 mm and 22.0 ± 2.2 mm × 18.5 ± 1.7 mm in diameter, respectively. MPIN 18G, with the needle array deployed 1 cm (MPIN-1cm), achieved a deposition measuring 23.3 ± 2.2 mm × 18 ± 3.5 mm. The same needle, with the array deployed MPIN-2cm, resulted in three spherical-like gel depositions, each with diameters of 12.7 ± 0.3 × 11.0 ± 0.4 (n = 8).

Using the same needle devices, iodine injections of 40 mg/mL (without gel) were performed and showed a high degree of leakage of material to nearby blood vessels (Fig. 4c).

Experiments using the MPIN device required use of a high-pressure syringe, as standard plastic syringes deformed under the pressures exerted to force extrusion of the gel through the three 27G needles of the device. The MPIN was unsuitable for infusing POL at a 1000 mL/h rate because of syringe deformation. Therefore, further analysis did not include this condition.

Table 1 summarizes the ex vivo imaging calculated for a variety of injection parameters such as needle type and injection rate for iodinated POL22 segmented at 80 kVp. The volumes calculated for formulations with POL were close to the 4 mL theoretical value except for MPIN-1 cm infused at 100 mL/h. Infused formulations without POL had higher standard deviations in segmented volume. Overall, POL injections were more reproducible compared to direct iodine injections.

**Table 1:** Overview of parameters and results for 4 mL infused gels: needle type, infusion rate, mean radiopacity, calculated injected volume, surface area, and percent volume error.

Formulation	Needle type; injection rate (mL/h)	Mean radiopacity (HU)	Calculated volume from segmentation (mL)	Surface area (cm^2^)	% Error of volume
POL22 + Iodine	SEHN; 1000	828.1 ± 39	4.8 ± 0.6	15.1 ± 1.6	16.2
	SEHN;100	822.2 ± 124.9	5.3 ± 0.3	17.2 ± 1.0	25.3
	SEHN; 10	892.6 ± 89.9	4.3 ± 1.4	13.9 ± 2.4	6.7
	MSHN; 1000	321.5 ± 31.8	3.9 ± 0.1	13.4 ± 0.6	2.5
	MSHN; 100	881.1 ± 145	4.8 ± 1	15.1 ± 1.7	16.7
	MSHN; 10	856.2 ± 34.6	4.79 ± 0.3	14.7 ± 0.7	16.5
	MPIN-1cm; 100	724.4 ± 83.3	2.3 ± 0.8	10.4 ± 3	72.8
	MPIN-1cm; 10	732.4 ± 164.8	3.6 ± 0.6	15.5 ± 0.9	9.8
	MPIN-2cm; 100	700.1 ± 33.9	3.7 ± 0.4	20.3 ± 1.9	9.3
	MPIN-2cm; 10	525.9 ± 128.7	4.0 ± 1.2	22.8 ± 7.2	0.8
Free iodine	SEHN; 10	342.8 + 213.8	3 ± 2.1	22.6 + 15.9	35
	MSHN; 10	297.7 + 46.5	2 ± 1.8	19.3 + 17.9	102.2
	MPIN-1cm; 10	309.2 + 260.3	4.9 ± 2.6	37.1 ± 26.1	17.8
	MPIN-2cm; 10	378.8 + 28.3	6.6 ± 1.5	63.1 ± 8.6	39.6

**Figure 5:**
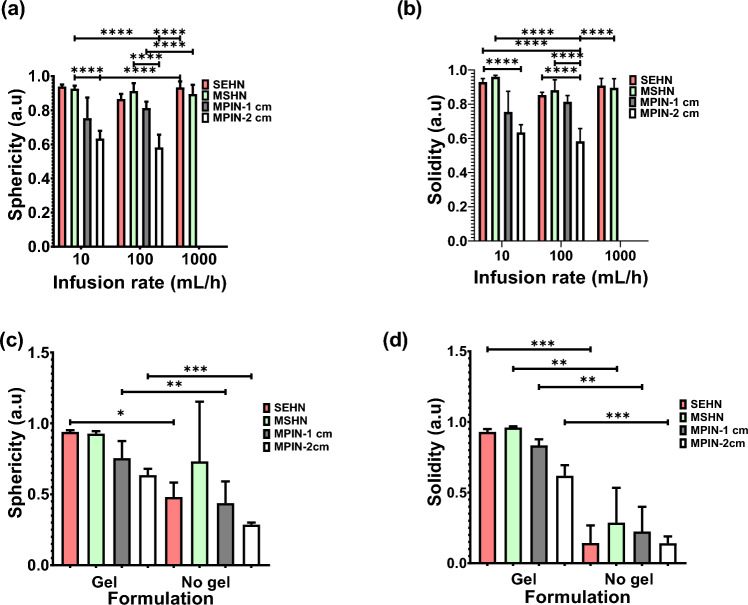
Morphometric analysis of POL22 with iodine injections into ex vivo bovine liver. (**a**) Sphericity of 4 mL injections as influenced by the infusion rate and the needle device utilized; n = 4 for SEHN at 1000 mL/h. (**b**) Solidity of 4 mL injections as a function of the infusion rate and needle device. (**c**) Sphericity of 4 mL injections at an infusion rate of 10 mL/h, compared with their free iodine counterparts, n = 4 for MSHN-no gel. (**d**) Solidity of 4 mL injections at 10 mL/h, compared with their free iodine counterparts, n = 4 for MSHN-no gel. Sample sizes were n = 3, except as noted. Error bars represent standard deviations of mean value. *p < 0.05, **p < 0.01, ***p < 0.001, and ****p < 0.0001, from one-way ANOVA statistical test.

To evaluate the morphometry of POL22 injections as a function of the infused volume, volumes of 4, 8.6, and 14 mL of the gel were injected at 1000 mL/h using SEHN and the resulting sphericities and solidities were calculated (Fig. S7). The sphericity and solidity of 4 mL of iodinated POL22 were evaluated for SEHN, MSHN, and MPIN needle devices at different injection rates (Fig. 5a and b). Sphericities across all needle devices and injection rates were compared and were generally close to one, except for the MPIN-2cm, as it produced three separate depositions (Fig. 5a). SEHN demonstrated sphericities of 0.9 ± 0.0, 0.9 ± 0.0, and 0.9 ± 0.0 at injection rates of 10, 100, and 1000 mL/h respectively. MSHN injections exhibited sphericities of 0.9 ± 0.0, 0.9 ± 0.0, and 0.9 ± 0 at 10, 100, and 1000 mL/h respectively. A 10 mL/h injection with MPIN-1cm produced a sphericity of 0.7 ± 0.1 and a 100 mL/h injection with the same needle presented a sphericity of 0.8 ± 0. For MPIN-2cm, the sphericity was 0.6 ± 0.0 at 10 mL/h and 0.6 ± 0.1 at the 100 mL/h injection rate. Similarly, solidities approximated one across all needle devices and injection rates, except for MPIN-2cm. SEHN showed solidities of 0.9 ± 0.0, 0.8 ± 0.0, and 0.9 ± 0.0 for 10, 100, and 1000 mL/h injection rates respectively. MSHN reported solidities of 1.0 ± 0.0, 0.9 ± 0.1, and 0.9 ± 0.0 for 10, 100, and 1000 mL/h. The solidity decreased to 0.7 ± 0.1 at 10 mL/h and 0.8 ± 0.0 at 100 mL/h for MPIN-1cm.

The sphericity and solidity of 4 mL POL22 gels injected at 10 mL/h were assessed across the three needle devices and compared with a free iodine (Fig. 5c and d) with decreased sphericity and solidity for iodine without gel. For instance, the sphericity and solidity of the gel-free formulation for SEHN decreased by 48.9% and 85% respectively in contrast to its free iodine formulation analogue. In the case of MSHN, the sphericity decreased by 21.5%, and the solidity by 70.8% compared to the POL22 formulation.

**Figure 6:**
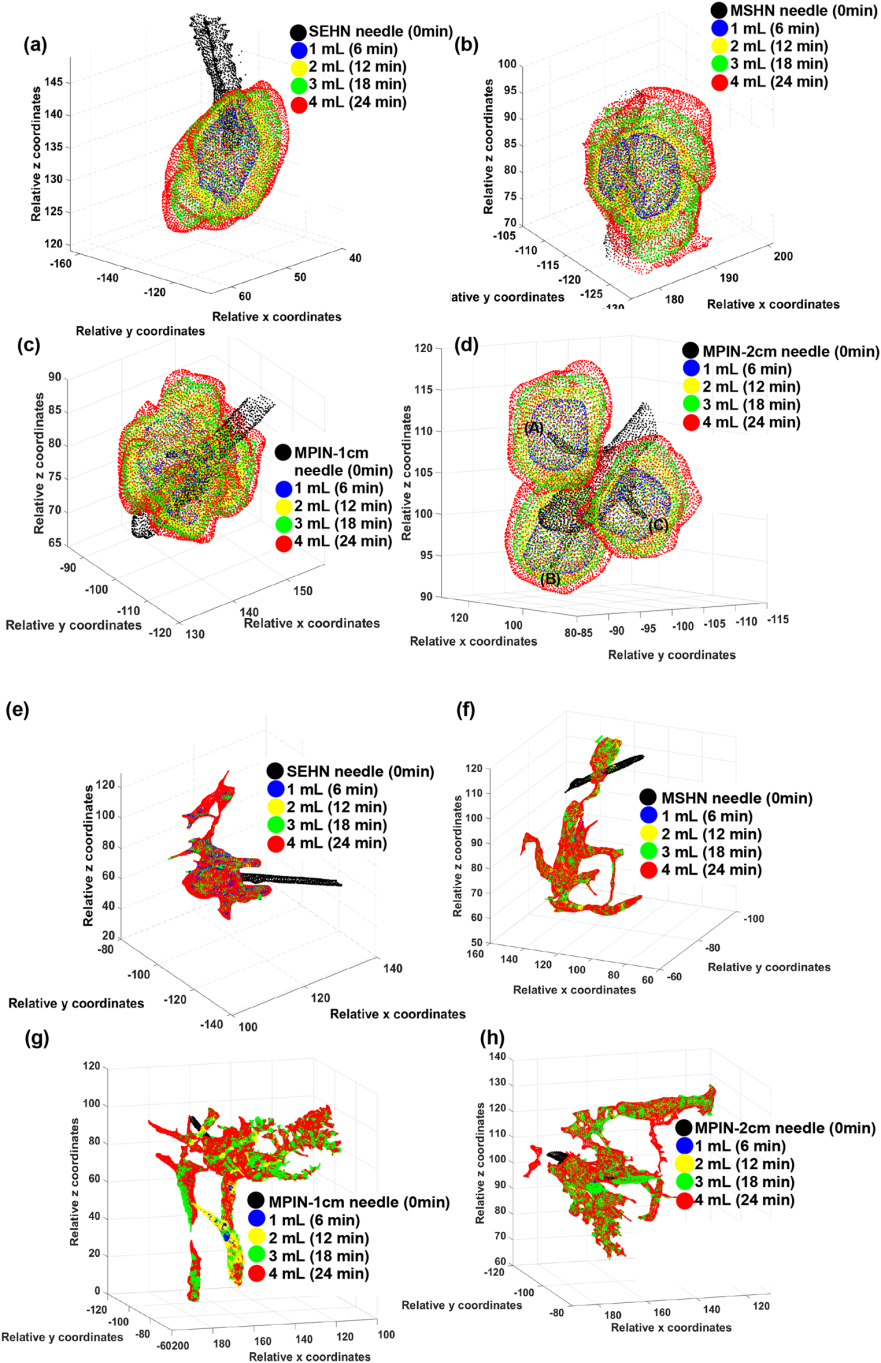
Three-dimensional temporal progression and volumetric distribution of needle injections, 4 mL at 10 mL/h in ex vivo bovine liver. The transparent surface renderings of the volumes at 1 mL increments (1, 2, 3, and 4 mL total injected volumes) based on cone beam CT across different needle devices are superimposed. (**a**–**d**) POL22 with iodine and (**e**–**h**) iodine alone. Four needles were used: SEHN (**a**, **e**), MSHN (**b**, **f**), MPIN deployed 1 cm (MPIN-1cm) (**c**, **g**), and deployed 2 cm (MPIN-2cm) (**d**, **h**) For POL22 with iodine utilizing MPIN-2cm (**d**), A, B, and C denote the three needle tips.

The 3D spatiotemporal distribution of the infused gel was evaluated across the three needle devices (Fig. 6a–d). All injections were localized post-injection in tissue with minimal vessel leakage. Each deposition after each incremental infusion of 1 mL exhibited high sphericity with rounded edges for both SEHN and MSHN (Fig. 6a and b). For the MPIN-1cm, the sphericity degree was less than that for SEHN and MSHN (Fig. 6c). Although leakage reduced these morphometric values, the separated gel depositions from each needle tip (A, B, and C) for MPIN-2cm were also spherical (Fig. 6d). The solidity of SEHN, and MSHN were also high compared to MPIN-1cm. For MPIN-2cm, the solidities were high for each deposition from each needle tip. In the case of iodine injection, the distribution was nonlocalized and irregular in shape, with a high degree of extravasated material (Fig. 6e–h). Table S3 summarize the sphericities and solidities for each needle device for quantitative assessment.

**Figure 7:**
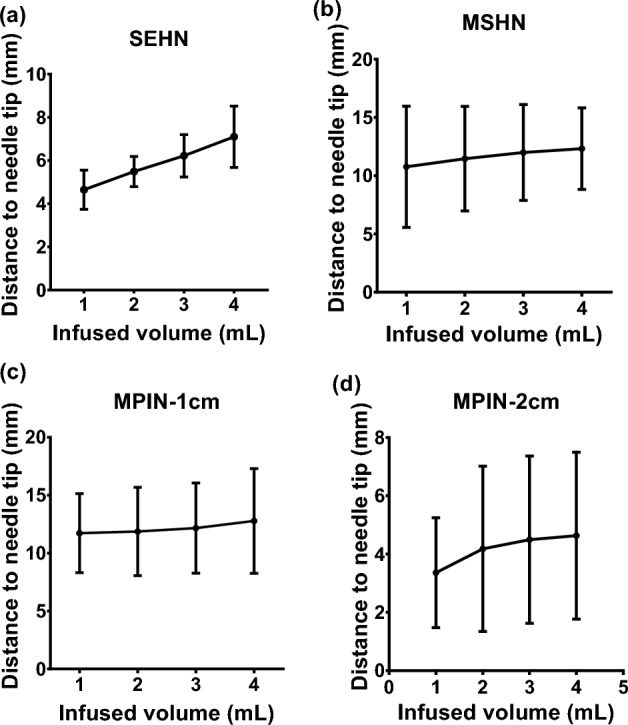
Relationship of injected POL22 with iodine to the needle tip. Measurement of the distance from the centroid of the injected POL22 with iodine (4 mL) to the needle tip in ex vivo liver, based on cone beam CT, for four needles: SEHN, MSHN, MPIN-1cm, and MPIN-2cm (**a**–**d**, respectively). Sample size of n = 3 for all needles, except for MPIN-2cm, n = 9. Error bars represent standard deviations of mean value.

To evaluate the predictability of the injections in 3D, the distance from the centroid of each injection to the needle tip for each 1 mL infused was calculated across the three needle devices, up to the total volume of 4 mL (Fig. 7a–d). The relationship between the distance (in mm) and the infused volume yielded a linear pattern for SEHN (y = 0.8x + 3.8, R^2^ = 1) (Fig. 7a), MSHN (y = 0.5x + 10.3, R^2^ = 1) (Fig. 7b), and MPIN-1cm (y = 0.3x + 11.3, R^2^ = 0.9) (Fig. 7c). For the MPIN-2cm device, a linear plots was produced (y = 0.4 + 3.1, R^2^ = 0.9) (Fig. 7d).This plot revealed a linear correlation between volume and distance to the center, except for one instance, which showed a deviation due to some degree of leakage.

**Figure 8:**
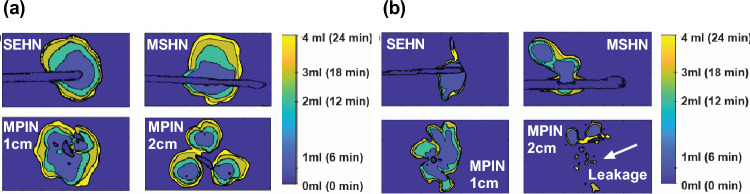
Two-dimensional spatiotemporal distribution over time of needle injections, 4 mL POL22 with iodine at 10 mL/h in ex vivo bovine liver. The surface contours of the volumes at 1 mL increments (1, 2, 3, and 4 mL total injected volumes) based on cone beam CT are superimposed. Four needles were used: SEHN, MSHN, MPIN deployed 1cm (MPIN-1cm) and deployed 2cm (MPIN-2cm) (**a**) POL22 with iodine. (**b**) Iodine alone. White arrow depicts leakage.

**Figure 9:**
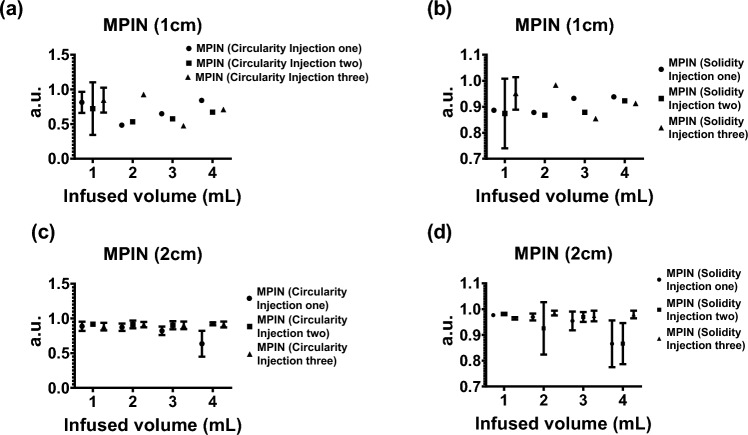
Circularity and solidity for POL22 with iodine, 4 mL injected at 10 mL/h in ex vivo bovine liver. The circularity (**a**, **c**) and solidity (**b**, **d**) based on cone beam CT for MPIN deployed 1 cm (**a**, **b**) and deployed 2 cm (**c**, **d**). Three injections were performed for each device and the values of circularity and solidity are reported for each injection at 1 mL increments (1, 2, 3, and 4 mL total injected volumes). Error bars represent standard deviations of mean value. n = 3 for all the experiments.

The 2D spatiotemporal gel dynamics in bovine tissue depicted a clearly outlined growth of the injected material across the three needle devices for a 4 mL injection at 10 mL/h (Fig. 8a). Formulations containing gel exhibited an expansion in the outlined area with each incremental 1 mL injected, as demonstrated by color coding. However, delineating the expansion of the injected material posed a challenge for formulations without gel across all three needle devices (Fig. 8b). The circularity and solidity of each gel deposition over time were analyzed for each injection (Table S4, and Fig. 9). SEHN and MSHN generated one deposition per mL injected (Table S4). For the case of MPIN-1cm (Fig. 9a and b), circularity and solidity values for each injection were reported separately because in some cases they produced more than one gel deposition per mL injected. The first 1 and 2 mL of injection resulted in multiple distinct areas due to the needle's three tips as separate points of origin, whereas for 3 and 4 mL, the material coalesced into a single gel deposition. Circularity measurements for 3 and 4 mL of infused material were 0.6 ± 0.1 and 0.7 ± 0.1 respectively, with solidity measurements of 0.9 ± 0.0 and 0.9 ± 0.0. For MPIN-2cm (Fig. 9c and d), each injection was reported separately since each needle produced three separate depositions. The average circularity for each deposition across the three injections was 0.9 ± 0.0 for 1 mL, 0.9 ± 0.0 for 2 mL, 0.9 ± 0.0 for 3 mL, and 0.8 ± 0.2 for 4 mL. The 4 mL injection result showed a higher standard deviation due to slight leakage to a vessel from one of the three tips during one of the injections. The average solidities (Fig. 9d) for each of the three depositions per incremental 1 mL were 1 ± 0.0 for 1 mL, 1 ± 0.0 for 2 mL, 1 ± 0.0 for 3 mL, and 0.9 ± 0.0 for 4 mL. Table S4 summarizes the sphericities and solidities for each needle device for quantitative assessment.

**Figure 10:**
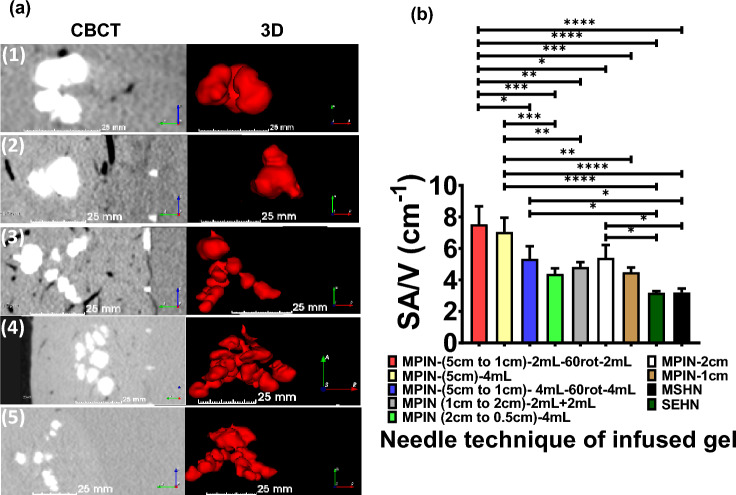
Distribution of POL22 with iodine, 4 mL injected at 10 mL/h, for MPIN injection techniques (1) through (5), as defined in the “Materials and methods” section, MPIN injection technique variations. (**a**) 3D surface renderings (left) and corresponding cross-sectional images (right) based on CBCT imaging. Distance scale bars are shown. (**b**) Surface area to volume (SA/V) ratios for each injection technique, *p < 0.01, **p < 0.006, ***p < 0.0003, ****p < 0.0001 from one-way ANOVA statistical test. For techniques 1, 2, and 4, n = 3; technique 3, n = 6; technique 5, n = 4. Error bars represent standard deviations of mean value. Technique 1—MPIN-(5cm to 1cm)-2mL-60rot-2Ml, Technique 2—MPIN-(5cm)-4mL, Technique 3—MPIN-(5cm to 1cm)-4mL-60rot-4mL, Technique 4—MPIN (1cm to 2cm)-2mL + 2mL, and Technique 5—MPIN (2cm to 0.5cm)-4mL.

Depending on the MPIN needle technique, the 3D morphology of the injected gel changed. Table S5 summarizes the obtained morphologies from the MPIN injections. Figure 10a depicts the morphology of various MPIN injection techniques. Depending on the MPIN needle technique, the SA/V ratio also changed for the techniques (Table S5) and the results were as follows: technique one (4.8 ± 0.3/cm), technique two (4.4 ± 0.3/cm), technique three (7 ± 0.9/cm), technique four (5.3 ± 0.8/cm), and technique five (7.5 ± 1.1/cm) (Fig. 10b). The results of SA/V from MPIN-1cm and MPIN-2cm were also calculated with values of 4.5 + 0.3/cm and 5.4 + 0.8/cm, respectively. 3D gel structures deposited in tissue with SEHN and MSHN devices exhibited SA/V ratios of 3.2 cm^-1^ that is akin to theoretical values corresponding to spherical shapes with 1 cm radius.

### Drug distribution in tissue

**Figure 11:**
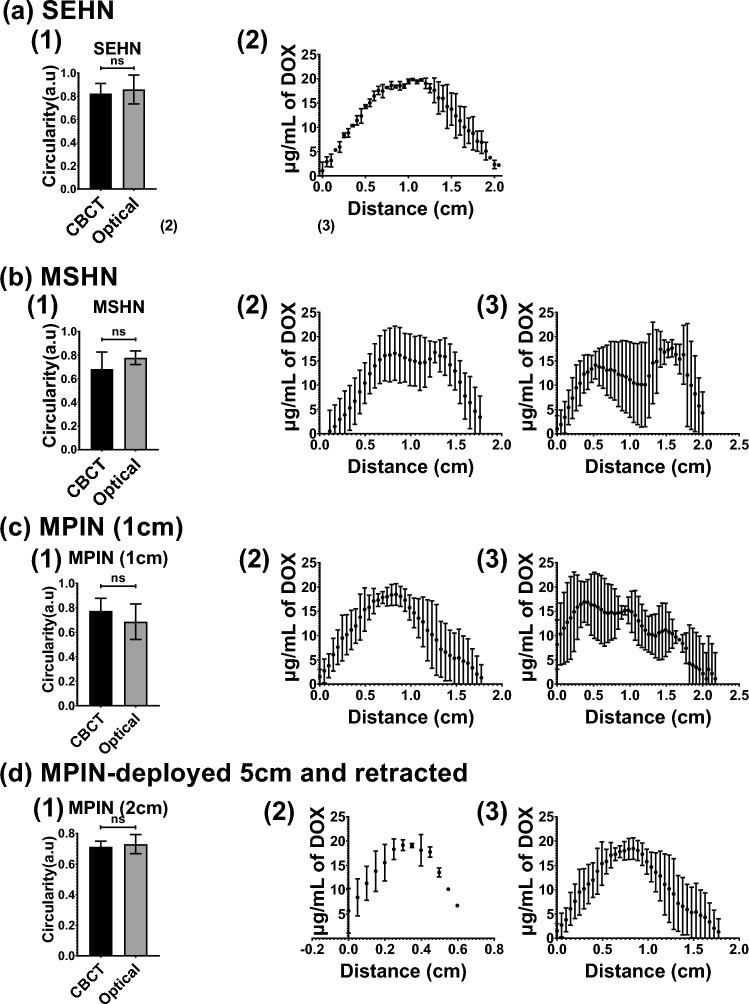

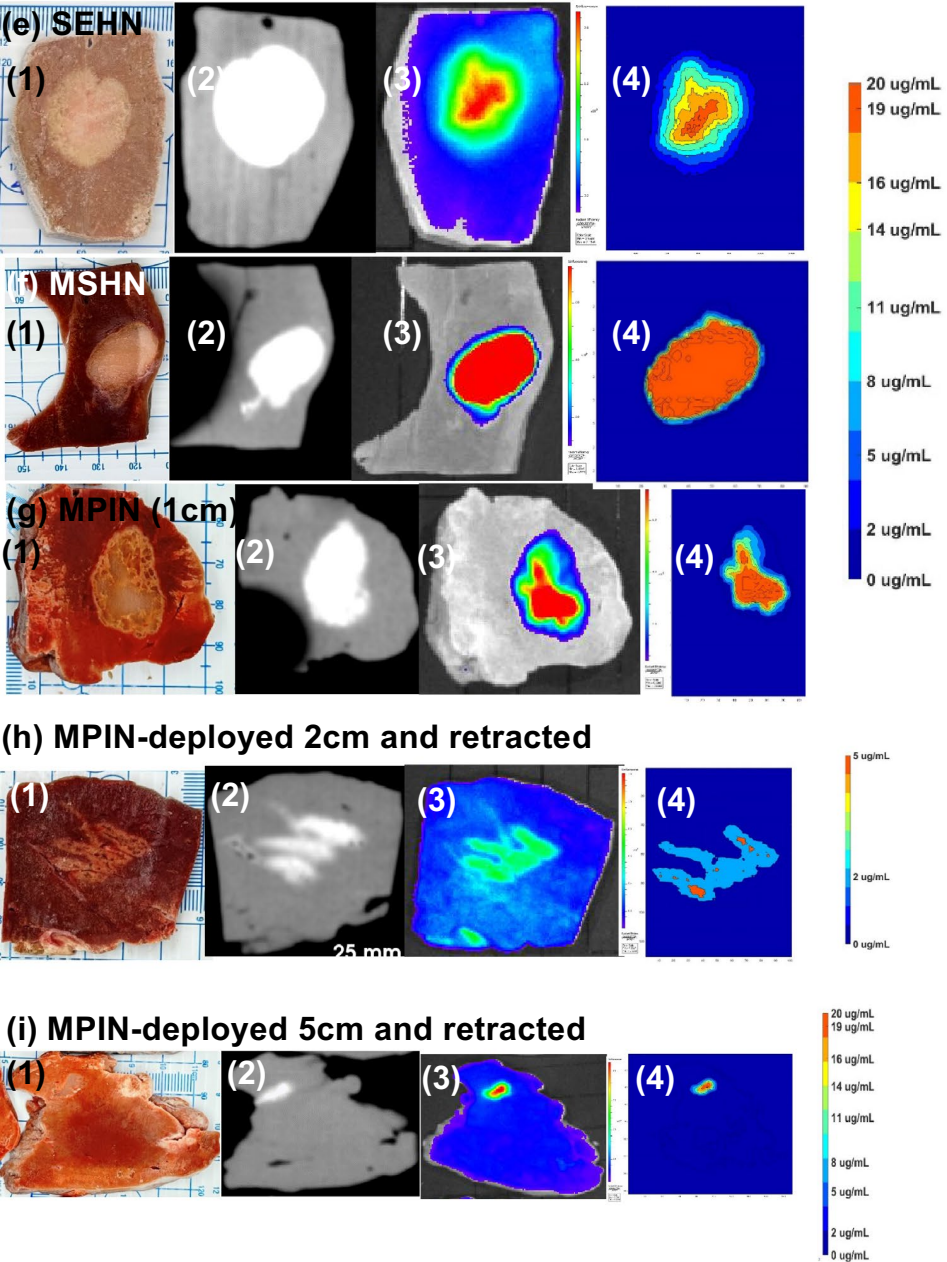
Comparison of cone beam CT (CBCT), optical imaging of DOX, and DOX drug mapping following injection of 4 mL POL22 with iodine and DOX, 20 μg/mL. Three devices were used: SEHN, MSHN, and MPIN. Three procedures were used for the MPIN: MPIN-1cm; MPIN deployed 2 cm and retracted during injection (technique 2); and, MPIN deployed 5 cm and retracted during injection (technique 5). (**a**–**d**, column 1) Circularity of optical imaging of DOX on the cut surface of the bovine liver compared to iodine on corresponding multiplanar reformatted sections on CBCT. (**a**–**d**) Relative concentration of DOX in µg/mL over the diameter for three infusions (column 2, minor axis; column 3, major axis). (**e**–**i**) Gross section (column 1), multiplanar reformatted sections on CBCT (column 2), DOX fluorescence intensity with optical imaging (column 3), and drug mapping (column 4). ns, not significant. Error bars represent standard deviations of mean value. n = 3 for all the experiments.

To investigate drug distribution in tissue, 4 mL of POL22 + iodine containing 20 µg/mL DOX were infused into bovine liver using SEHN, MSHN, and MPIN devices at 10 mL/h. SEHN injections exhibited circular patterns with optical and CBCT imaging circularity values both at 0.8 ± 0.1, indicating accurate targeting (Fig. 11a1). DOX concentration was highest at the center of the injection, decreasing towards the edges (Fig. 11a2). MSHN injections showed circularity of 0.8 ± 0.1 (optical) and 0.7 ± 0.1 (CBCT), with a ratio of 0.9, (Fig. 11.b1). Fluorescence intensity measurements across the injection site suggested a more even distribution (Fig. 11b2, and b3). MPIN-1cm provided similar circularity values of 0.7 ± 0.1 (optical) and 0.8 ± 0.1 (CBCT), in the plane of the needle tips with a 1.2 ratio, highlighting a varied drug distribution consistent with the multiple infusion points (Fig. 11c1–c3). A 0.2 mL gel deposition analysis, from injection technique 5, showed uniform distribution with circularity of 0.7 ± 0.1 (optical) and 0.7 ± 0 (CBCT), and a 0.98 ratio of circularity (Fig. 11d1–d3). Figure 11e–i illustrates tissue samples, CBCT images, fluorescence imaging, and drug maps, showing qualitative correlation and varied distribution profiles per device.

### Gross pathology of injected material

**Figure 12:**
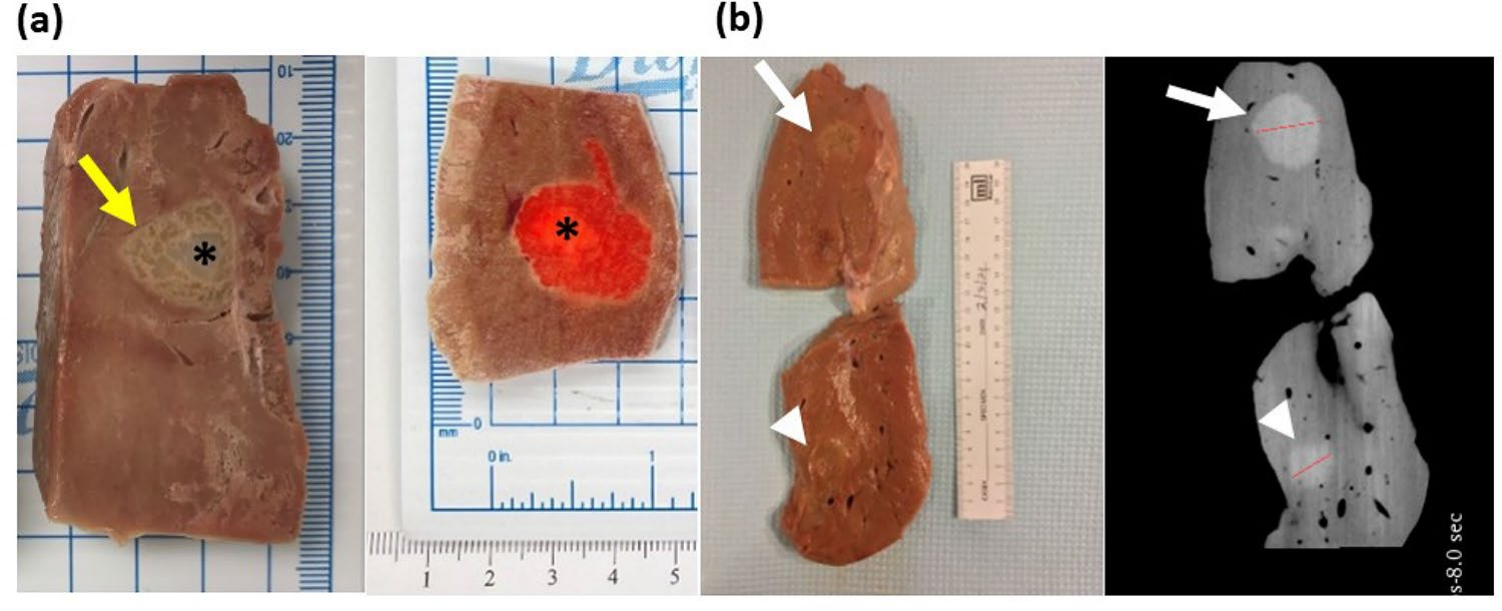
Gross pathology and imaging of bovine liver frozen following injection of POL22 with and without DOX. (**a**) Cut liver showing injections of POL22 with iodine, without DOX (left) and with 2 mg/mL of DOX (right). The injection core (asterisk) and gel extending into liver tissue (yellow arrow) are shown. (**b**) Gross image of the tissue section (left) and corresponding cone beam CT image (right) for 4 mL (top, white arrow) and 14 mL (bottom, arrow head) injections of POL22 with iodine but without DOX.

The gross examination of the liver after gel injection shows a gel core with gel permeating into the hepatic tissue (Fig. 12a). This distribution was similar for the gel containing 2 mg/mL of DOX, where the infused material localized primarily in the center. The diameters of the material following injection of 4 and 14 mL of hydrogel were 2.2 mm and 3.2 mm, respectively (Fig. 12b). These observations were further corroborated by CBCT imaging (Fig. 12b). These results confirm the high degree of localization of gel with drug and therefore its predictability post-injection.

## Discussion

This study investigated a poloxamer-based hydrogel, radiologically visible, as a drug delivery system. The gel was able to be injected in liquid and solid form and showed slow release in vitro. It was demonstrated, ex vivo, the importance of injection parameters for precise gel deposition and predictable drug deposition. Gels were delivered in a manner similar to that expected in clinical application, i.e., with image-guided needle insertion to the target location and then gel injection monitored with clinical imaging techniques.

The gelation time and temperature varied principally with the concentration of poloxamer with only modest variations with the addition of the studied concentrations of iodixanol and doxorubicin. This permits rational selection of the formulation since the gel needs to be capable of free flow with low viscosity while being injected and then, ideally, rapid gelation in situ^31,32^. Low viscosity after injection in vivo would permit undesirable movement or redistribution of the gel and drug away from the target delivery area and extravasation into surrounding vessels^31^. The selected formulation, POL22, can be delivered as liquid or as gel by syringe and will form gel at body temperature once injected into an organ or tumor due to its thixotropic properties as shown from the rheological analysis. Even if the injection path or injection rate were to lead to exposure of the gel to body temperature for a prolonged period, injection could still be performed as the gel viscosity is reduced under high stress, i.e., during injection, and recovers its high viscosity properties once the stress is removed. Gelation time could be titrated with changes in POL content to meet specific needs or titrate conformality according to specific drug mechanism or target.

From the in vitro elution profiles, it can be inferred that direct injections of iodine and DOX without gel would be fleeting due to rapid distribution and tissue perfusion. The use of POL22 as a drug carrier, should lead to slower release kinetics compared to DOX solutions. This was demonstrated with ex vivo injections of iodine or POL22 with iodine into bovine liver. This reduction in release may increase the biological residence time of the drug at the targeted treatment region and serve as a drug reservoir, such that the therapeutic dose will remain high over a longer time period compared to the direct injection of DOX under perfusion conditions^33^. In clinical settings, this could allow reduction of repeated drug doses due to its sustained release kinetics, while also reducing systemic exposure to acute peaks of toxic agents^34,35^. Although in vivo interstitial and intravascular pressure and flow may alter results, the free-flow behavior of free iodine injection observed ex vivo supports the concern regarding risk of nonlocalized drug deposition and therefore burst release in vivo^36^ with resulting systemic exposure compared to localized injections with gels. The characteristics of injectability and gel localization of iodinated POL22 met the clinical requirements for localization, predictability, and reproducibility^13^. The high localization of iodinated POL22 gels is attributed to their rapid G’ recovery from liquid-like to a gel-like state post-injection and their fast gelation kinetics at physiological temperature.

There may be differences in drug release rates from the gel within tumor tissue compared to normal tissue, as the acidic pH in tumors will affect the hydrophilicity of the polymer disrupting the formed micelles^37^. These polymer and aqueous media interactions will result in more rapid dissolution of POL and consequent drug release within the tumor^38^. Gonzalez et al.^39^ reported that formulations containing POL and other polymers had 45% drug release in distilled water, 57% in 0.1N HCl and 53% in PBS.

The drug efficacy will depend on the drug and its specific pH-dependent interactions with tissue. For the specific case of DOX, it has been shown that the low extracellular pH in tumors will reduce the DOX (weak base) uptake^40^. This may limit bioavailability to tumor, but is an issue for all methods of DOX delivery to tumors.

The degradation profile and biodistribution of POL, particularly for intratumoral delivery, are still areas to explore further. Chatterjee et al.^37^ reported POL degradation at pH 7.4, and 37 °C in PBS which showed a remaining mass of 39.2% after 14 days. Few reports have shown that POL accumulates in hepatic tissue more than in the kidneys, in addition, the half-life of elimination of poloxamer 407 in rats after intraperitoneal injection has been reported to be 20.9 h^41^.

The addition of iodixanol (iodine) permitted imaging of the injection and gel distribution. Suitable concentration of iodine to permit imaging with CBCT and fluoroscopy was determined. As with DOX, elution of iodine from the gel was prolonged compared to a simple solution, suggesting that the initial distribution of the gel could be matched to that seen on imaging. This was confirmed with CBCT imaging of POL22 with iodine and fluorescence imaging of DOX showing congruent distribution. While differences in the drug and iodine diffusion within the gel, transport into tissue, and tissue pharmacokinetics may lead to loss of co-localization of drug and iodine over time, the initial deposition can be defined, meeting a key development goal. The unpredictability of direct percutaneous delivery of free iodine seen in this study is similar to the clinical challenge encountered during intratumoral injections with high volumetric variability among injection parameters^28^. The localization of infused material such as free iodine can be also impacted with the needle type and injection technique. For example, use of the MPIN had more localized distribution in tissue, as evaluated clinically^28,42^ and preclinically^29^. However, the presence of multiple needles increases the risk of direct intravascular administration.

The high degree of sphericity observed for injected POL22 with iodine, regardless of infusion rate, suggests the potential for creating localized treatment zones or volumes of drug deposition. High solidity values close to one for both SEHN and MSHN indicate 3D uniformity, which may result in more predictable and controllable treatment zones. After delivery, measures of sphericity and solidity may help to quantify treatment zones, margins, and localization. Across the three needle types, the injected gel formulation maintained an individual but consistent configuration which allows for a defined morphology injection with each needle device. The clearly distinguishable boundaries of the growing material during injection offer spatial predictability when considering potential treatment zones per milliliter injected.

Having information such as distance to the needle tip and 3D volumetric distribution might influence planning of needle tip placement or drug delivery, and inform any required intraprocedural positioning^43^. For example, the confirmation of needle positioning, necessary adjustments, and even needle advances can be verified through CT imaging. Despite the presence of artifacts due to the metal content of the needles, CT with metal artifact reduction tools and registration with pre-operative imaging offers a reliable means of visualizing the needle's position and allows for a sequential evaluation of gel treatment. These techniques can be incorporated into clinical workflows^44,45^, either with CT imaging alone or in combination with other modalities such as ultrasound for image fusion applications^45–48^. Such workflows may include planning, targeting, monitoring, and making intraprocedural iterative modifications^45^.

In general, lower volumes will have higher dose control compared to larger volumes. The surface-area-to-volume ratio (SA/V) can be modulated according to the specific injection technique used, particularly with the MPIN. The myriad locations and patterns in which gel can be injected by varying the needle depth and the manner in which MPIN is rotated can significantly change this ratio, even with a constant injection volume. This shows the potential for tailored treatment zones based on device configuration and drug delivery method. The differences in SA/V may impact drug pharmacokinetics by virtue of the surface areas available for drug flux from the gel as well as potential for differing rates of POL biodegradation and related drug release. The potential to control physical location of drug and the SA/V may allow refinement of delivery to meet target pharmacokinetics parameters for a selected drug. The ability to tune drug distribution with needle devices and techniques suggests suitability for different tumor shapes, enhancing drug delivery precision.

There are advantages and disadvantages for each of the tested needles. The SEHN provided a predictable, defined delivery of gel due to its single hole at the end of the needle. However, the SEHN had limited tunability and morphological control. It has been reported in preclinical studies that MSHN provided better drug distribution compared to SEHN. One limitation of the MSHN is that the deposition of the gel will depend on the location of all the side holes as sources of delivery. Insertion of the needle through a vessel could be an issue if a side hole opened into the lumen. The major advantage of MPIN is the morphological tunability that can be controlled by the separation between the needle tips, staged needle retractions during injection, and rotation of the central needle and redeployment of the prongs with further injection. However, the MPIN required higher injection pressures during POL delivery due to the small needle lumens. Therefore, the use of high-pressure syringes or devices to deliver the drug may be required. In addition, gel delivery and localization will depend on the localization of each of the needle tips, e.g., proximity to vessels, as well as injection rates and intrinsic properties of the target tissue. Furthermore, since some malignant tissues have interstitial pressures higher that normal^49,50^, tumoral injection may require higher injection pressures with concomitant use of high-pressure syringes and connector tubing. Interstitial pressure heterogeneities should be a determinant of distribution. This will be worthy of further investigation prior to clinical translation.

DOX was used as an example drug as its use in locoregional delivery is widespread and its spatial distribution can be easily characterized. In translation to clinical applications, we anticipate use of the gel with other molecules such as immunomodulatory drugs or monoclonal antibodies, potentially providing therapeutic benefits without the dose-limiting systemic toxicities^20^. Ning, et al. reported use of a collagen gel for delivery of radiolabeled antibodies for radioimmunotherapy which improved tumor uptake, increased therapeutic index and reduced systemic toxicity^51^. Another study using POL loaded with CTLA-4 checkpoint inhibitor also demonstrated reduction of antibody levels and slowed down tumor growth^20^.

There are several potential benefits to intratumoral drug delivery compared to intravenous administration^1,34,52^. Overall pharmaceutical costs may be lower as less drug is required compared to systemic intravenous administration, Gel-based delivery also shares the inherent advantages of other locoregional therapies in that higher doses may be locally delivered directly to the tumor compared to systemic delivery with attendant reduction in systemic side effects. Direct intratumoral injections may allow also multiple injections at the target. Incorporation of drugs into gels may allow prolonged local administration as the drug elutes from the gel. Intratumoral injections may increase drug efficacy by minimizing off-target delivery which may mitigate drug efficacy and safety^12,28^.

There were limitations to the study. The testing of tissue injection was conducted in normal bovine liver without blood flow or tissue perfusion biology that would affect drug kinetics. There will be variability in cancerous tissue with differences in mechanical properties among tumor types compared to normal tissue which may affect delivery and distribution. However, a main goal of the study was to define a contextual background for development of POL as formulation not to thoroughly verify deposition rather than pharmacokinetics following delivery. Further, the proposed application of the drug-eluting gel is for clinical cancer therapy where the tissue properties of diverse organ tumors, including physical properties and the biochemical milieu, will differ from normal liver and will be unique to the tumor in different organs. This will require granular characterization of POL in tumor mimicking tissues. As next steps in evaluation, in vivo pharmacokinetics studies can be used to demonstrate the improved locoregional delivery with reduced systemic exposure. In addition, the effects of tissue disruption during and after injection need to be systematically studied to evaluate micro and macro consequences of tissue displacement, including local drug distribution and impact on metastatic potential. The interplay of injection pressures and the adjacent tissue interstitial pressure exerted into the deposited gel also needs to be studied in vivo to evaluate the pressure component on drug distribution in tumors. Next steps in translation might include study in tumor-mimicking phantoms, animal models of cancer, or potentially surgical explants of tumors, morphometrical characterization of gel delivery in different tissues with different spectrums of mechanical properties, or specific drug selection with pharmacokinetics and biological characterization, all prior to potential clinical study.

## Conclusions

This study provides characterization of poloxamer-based hydrogels for injections in the liver. The x-ray imageability of the hydrogel allows for real-time observation and precise control during image-guided procedures. Cone beam computed tomography (CBCT) enabled accurate monitoring of a predictable and localized delivery of therapeutic agents, such as DOX, in an ex vivo bovine liver model. The 3D distribution of the hydrogel is controllable by needle selection, injection parameters, and techniques which represent a substantial improvement over traditional methods that inject low viscosity materials. This study emphasizes the 3D controllability of the hydrogel, including the ability to modulate the surface-area-to-volume ratio (SA/V). Controlling SA/V enables the modulation of therapeutic effects within targeted treatment zones. The precision and predictability in delivering potential anti-cancer agents has potential to minimize systemic side effects. This approach promises to improve treatment planning, reduce variability in drug delivery, and foster a higher degree of standardization. The strong correlation between optical imaging and CBCT underlines the feasibility of using x-ray-based imaging for planning and predicting drug distribution, to promote controlled feedback for procedures that otherwise suffer from variability. This research elucidates the critical interplay between injection techniques, imaging technology, and hydrogel properties, while seeking new standards for precision in image-guided localized drug delivery.

## Materials and methods

### Preparation of x-ray-imageable and nonimageable poloxamer-based hydrogels

X-ray-imageable Poloxamer 407 (POL) (Sigma Aldrich, St. Louis, MO, USA) formulations were prepared according to the cold method previously described by Huang et al.^27^, with modifications to generate five concentrations of POL, 17, 18, 20, 21, and 22% (w/v) (POL17, POL18, POL20, POL21, and POL22, respectively), in normal saline (Quality Biological, Gaithersburg, MD, USA) with iodixanol (Visipaque 320 mg I/mL, GE Healthcare (Boston, MA, USA) added to a final concentration of 40 mg I/mL. Briefly, the respective amounts of poloxamer, iodixanol, and normal saline were added to a total volume of 50 mL. The solution was maintained at 4 °C under magnetic stirring for at least 12 h. A similar procedure generated nonimageable POL formulations at the same poloxamer concentrations without iodixanol.

### Selection of iodine concentration for CBCT and fluoroscopy imaging

Serial dilutions of iodixanol in saline were prepared at 0.25, 0.5, 1, 2, 4, 8, 16, 32, 40, and 64 mg I/mL in 2 mL vials^53^. The samples were suspended inside a cylindrical water phantom to mimic x-ray attenuation in patients and imaged with CBCT and fluoroscopy (Allura Xper FD20, Philips, Best, the Netherlands)^53^. CBCT images were acquired with clinical imaging protocols at 120 kVp, 148 mA and 100 kVp, 184 mA. Cylindrical regions of interest (ROI) of 1mm radius and 8.265 mm height were used and segmented for each dilution in the CBCT, and attenuation in Hounsfield units (HU) was measured using open-source software (3D Slicer, URL https://www.slicer.org/). A linear regression analysis was performed to determine the relationship between CT numbers (HU) and mg I/mL, where a higher slope represents greater sensitivity to the detection of iodine^54^. The contrast-to-noise ratio (CNR) was calculated with a detectability threshold defined by the Rose criterion as CNR of 2.5 (Eq. 1)^55^ where µ_vial_ and µ_background_ are the HU values in the test solutions and background, respectively while σ_viaL_ and σ_background_ are the standard deviations. The iodine detectability threshold was used to determine the iodine concentration range that will be visible^53,56^. Fluoroscopic images of the vials were acquired using the same cylindrical phantom.

1 $$ {\text{CNR}} = \frac{{\mu_{vial} - \mu_{{{\text{Background}}}} }}{{\sigma_{Background} }} $$

### Gelation times

The gelation times of the imageable and non-imageable gels were qualitatively estimated via the tube inversion method^20,57^. Briefly, 400 µL of the POL preparation at 4 °C was added to 8 mL glass vials (Duran Wheaton Kimble, Millville, NJ, USA). The material was allowed to equilibrate at room temperature for 5 min and then the tube was submerged in a 37 °C water bath and periodically inverted. The time after water bath submersion at which the formed gel remained at the bottom of the glass vial was recorded.

### Oscillatory Rheology

Gelation temperature and viscoelastic properties of the materials were characterized using a Discover HR20 rheometer (TA Instruments, New Castle, DE) equipped with a 25.0 mm stainless steel parallel plate geometry with 500 µm gap. To prevent drying artifacts, mineral oil (ASTM oil standard) was applied to the geometry and stage with all the samples prior analysis.

#### Gelation temperature

The effects of gel concentration (%, w/v) and iodixanol on the gelation temperature (T_gel_) were determined. POL formulations (17, 18, and 22%, w/v) with and without 40 mg I/mL were subjected to a temperature ramp from 5 to 37 °C according to experimental parameters adapted from Baloglu et al.^58^. The samples were subjected to 0.2% strain and 6.0 rad/s angular frequency. Herein, we report the gelation temperature according to literature convention^59,60^. The gelation temperature is the midpoint storage modulus value taken from temperature-sweep rheological data that measures a free-flowing poloxamer colloid transitioning to the gel state. T_gel_ was also determined for POL22 containing 2, 5, and 10 mg/mL of DOX and iodine.

#### Viscoelastic properties

The viscoelastic properties and thixotropic behaviour of POL gel formulations (17,18, and 22%, w/v) with and without 40 mg I/mL were determined with a time sweep experiment monitoring the G′ and G″ for 1 h at 0.2% strain and 6 rad/s at 37 °C. Then, a 1000% oscillation strain was applied for 1 min followed by 60 min at 0.2% strain to evaluate the recovery of the materials. The percent recovery was calculated from the plateau G′ value before high shear strain and the recorded G′ value at 0, 10, and 56 min post-shearing. Frequency sweeps were performed at 0.2% oscillation strain from 0.1 to 100 rad/s, and amplitude sweeps at 6 rad/s were performed from 0.1 to 1000% oscillation strain to determine the linear viscoelastic regions and flow points of the materials. The flow point was calculated as the modulus crossover point (G′ = G′′, tan δ = 1) with the software TRIOS (TA instruments) using the cubic/linear method. The same methodology was used to evaluate the viscoelastic behaviour of POL22 containing 40 mg I/mL iodine and 2, 5, and 10 mg/mL of DOX.

### In vitro elution profiles of x-ray contrast agent and doxorubicin

In vitro iodine release kinetics of POL formulations (17, 18, and 22% (w/v)) with 40 mg/mL were obtained by filling a dialysis cassette (Pur-A-Lyzer Midi 3500, Sigma Aldrich) (3.5 kDa molecular weight cut-off) with the POL formulation and incubation in a shaker (Roto-Therm Plus, Ward’s Science, Rochester, NY, USA) under constant shaking (rocking mode, 10) at 37 °C in 40 mL of normal saline. Aliquots were taken at 0, 1, 2, 3, 4, 5, 6, 7, 21, 45, and 69 h with volume replacement. Absorbance was measured at λ = 281 nm^61^ with a Cell Imaging Multimode Reader (Cytation 5 Bio Tek, Agilent) and compared to an iodixanol standard calibration curve. Following the same procedure, the DOX concentration from POL22 with 2, 5 and 10 mg/mL of drug was calculated from absorbance measurement at λ = 483 nm with a calibration curve.

### Ex vivo gel injection

#### Injection needles

Three needle types were studied: an 18G, 10 cm needle with a single-end hole (single-end hole needle, SEHN, Chiba biopsy needle, Cook Regentec, Bloomington, IN, USA); a 19G, 7.5 cm needle with multiple holes on the side of the needle shaft (multiple side hole needle, MSHN, ProFusion Therapeutic Injection Needle, Cook Regentec, Bloomington, IN, USA); and, two variants of a device with an 18G needle capable of deploying three curved injection prongs at variable distances. The first could deploy a maximum of 2 cm (short tip, ST) while the second had a deployment range of up to 5 cm (regular). These are collectively referred to as the multi-pronged injection needle (MPIN, Quadra-Fuse, Rex Medical, Conshohocken, PA, USA).

#### Temperature control

Bovine livers (Balducci’s Market, Bethesda, MD, USA) were acquired fresh, transported briefly at ambient temperature, and then stored at 4 °C before use. Livers were typically used within one day of acquisition, but not more than two days. Livers were submerged in 10 L of 1× phosphate buffered saline at 37 °C and allowed to equilibrate. The temperature was controlled by a calibrated Ink Bird Tech thermocouple (Ink Bird Tech Shenzhen, China) and a Ktopnob heating coil (Ktopnob Silver Spring, MD, USA). To ensure the tissue reached 37 °C, two thermocouples were inserted at different positions within the liver as previously reported^62^.

#### CBCT and fluoroscopic imageability and injectability assessment of poloxamer 407 gels

POL22 with 40 mg I/mL, was injected into an ex vivo bovine liver previously heated to 37 °C under submersion in PBS 10×, 7.4 pH (Gibco Thermo Fisher Scientific, Waltham, MA, USA). Before each injection, a CBCT scan was conducted to locate potential injection sites in the liver that were relatively devoid of hepatic vessels. Once these sites were identified, the needle was connected to silicon tubing (Smith Medical ASD, Dublin, USA), and a syringe (Monoject, Dublin, OH, USA) which was preloaded with the gel and maintained on ice. A 12 mL syringe filled with gel was used for SEHN and MPIN, while a 6 mL high pressure syringe (Medallion, Merit Medical, South Jordan, UT, USA) was employed for the MPIN needles. Prior to attachment, the gel was advanced to the needle tip, after which the syringe was connected to a programmable injection pump (Harvard Apparatus PhD Ultra Syringe Pump, Holliston, MA, USA) prior to needle insertion.

Following insertion of the needle, its position within the liver was verified using CBCT. The threshold volume that could be injected without causing extravasation in the ex vivo livers was determined by evaluating injections of 4 mL, 8.6 mL, and 14 mL using the SEHN at an injection rate of 1000 mL/h. These target volumes correspond to the volume of spheres with diameters of 2 cm, 2.5 cm, and 3 cm, respectively.

Once the critical volume threshold preceding gel extravasation was identified, 4 mL injections at 10, 100, and 1000 mL/h were performed to examine the impact of injection parameters on the morphology of the gel deposit. A series of 4 mL control injections of saline (no gel) with 40 mg I/mL at 10 mL/h were also conducted. CBCT imaging was performed. The mean radiopacity and volume of the segmented iodinated injected gel were calculated. The percent of volume error was calculated using Eq. (2). For subsequent analysis, the gel morphologies were segmented and exported as stereolithography (STL) files.

2 $$ {\text{\% of volume error}} = { }\left| {\frac{theoretical \;volume - experimental \;volume}{{theoretical \;volume}}} \right| \times 100 $$

Table 2 provides a summary of the ex vivo liver injection parameters investigated with CBCT imaging.

**Table 2:** Summary of injection parameters with different needle devices: single end hole needle (SEHN), multiple side hole needle (MSHN), and multi-pronged injection needle (MPIN).

Formulation	Needle type	Volume	Injection rate	Gauge
Image-able Gel: POL22, 40 mg I/mL	SEHN	4, 8.6, and 14 mL	1000 mL/h	18
	SEHN	4 mL	10, 100, and 1000 mL/h	18
	MSHN		10, 100, and 1000 mL/h	19
	MPIN (deployed 1 cm and 2 cm)		10, 100, and 1000 mL/h	18
No Gel: Normal saline, 40 mg I/mL	SEHN	4 mL	10 mL/h	18
	MSHN		10 mL/h	19
	MPIN (deployed 1 cm and 2 cm)		10 mL/h	18

#### Morphology of injected gel

The sphericity and solidity of the gel injections, as outlined in Table 1, were calculated using 3D Slicer and Blender (Blender Foundation, Amsterdam, Netherlands). The equations employed to assess solidity and sphericity are as follows:

3 $$ {\text{Solidity}} = { }\frac{V}{{Convex\;{ }hull\; volume}} $$

4 $$ Sphericity = { }\frac{{\pi^{\frac{1}{3}} \left( {6V} \right)^{\frac{2}{3}} }}{SA} $$

‘V’ represents the volume and ‘SA’ signifies the surface area for the sphericity calculation. 3D Slicer was used to determine the SA and V of the infused material. Blender software was utilized to calculate the convex hull volume, which is defined as the smallest convex volume set that encompasses the previously segmented volume of the gel injections, after importing the STL file to the Blender software.

### Time course of gel Injection volume and distribution

The spatiotemporal volumetric distribution of the gel was examined by injecting 4 mL of POL22 with 40 mg I/mL into an ex vivo liver at 10 mL/h. CBCT scans (100 kVp) were acquired at t = 0, and at 6, 12, 18, and 24 min during the injection, corresponding to 0, 1, 2, 3, and 4 mL of injected volume, respectively. The SEHN, MSHN, and MPIN devices were assessed, with the MPIN deployed at both 1 and 2 cm. Gel volumes were segmented at each time point and exported as a STL file. Afterwards, the volumes were displayed as 3D color-coded distributions per mL injected using MATLAB (R2020a version, Mathworks, Natick, MA).

Using the segmented volumes per mL injected, 2D cross-sections were created by importing the STL files to Meshmixer software (Autodesk Inc. (2019) Autodesk Meshmixer. http://www.meshmixer.com), generated at each timepoint for each needle device. For SEHN, using the cutting tool in Meshmixer, planes were generated longitudinal to the needle axis and the same procedure was applied for MSHN adding an orthogonal plane. For MPIN, an orthogonal plane was generated with respect to the tip of each of the three needles. Each 2D section was manually converted to grayscale using Paint software (Microsoft, Redmond, WA) for subsequent conversion to color contours with a customized code in MATLAB.

The sphericity (Eq. 3) and solidity (Eq. 4) for each injected volume were calculated. The circle-like shape (circularity) and determination of convex or concave shape (solidity) for the 2D cross-sections were also computed (MATLAB).

The centroid point [P_c_ = (x_c_, y_c_, z_c_)] of the 3D distribution after each milliliter injection was computed using Blender. The distance (d) between the centroid and the needle tip [P_n_ = (x_n_, y_n_, z_n_)] was calculated using the formula:

5 $$ {\text{d }}\left( {{\text{P}}_{{\text{c}}} ,{\text{ P}}_{{\text{n}}} } \right)\, = \,\sqrt {\left( {\left( {x_{n} - x_{c} } \right)^{2} + \left( {y_{n} - y_{c} } \right)^{2} + \left( {z_{n} - z_{c} } \right)^{2} } \right)} $$

### MPIN injection technique variations

The volumetric distribution and surface-area-to-volume (SA/V) ratio of MPIN injections were characterized for five variations in technique. All injections used POL22 with 40 mg I/mL at 100 mL/h.

1. Using a short tip needle, the prongs were deployed 1 cm, i.e., the distance between the tips was 1 cm (MPIN-1cm). After incremental injection of 2 mL, the prongs were advanced to 2 cm for the remaining volume of 2 mL, without rotation of the device.
2. Using a short tip needle, the prongs were deployed 2 cm (MPIN-2cm). During the injection of 4 mL, the prongs were retracted in four 0.5 cm increments from 2 to 0.5 cm, without rotation of the device.
3. Using a long tip needle (“regular”), the prongs were deployed 5 cm. During the injection of 4 mL, the prongs were retracted in five 1 cm increments from 5 to 1 cm. Approximately 0.8 mL were injected per deployment increment, without rotation of the device.
4. Using a long tip needle (“regular”), 4 mL were injected as defined in technique 3. The prongs were then withdrawn, the needle rotated 60°, and the prongs redeployed to 5 cm. Another 4 mL was then injected in the same manner for 8 mL total.
5. Using a long tip needle (“regular”), 2 mL were injected as defined in technique 3. The prongs were then withdrawn, the needle rotated 60°, and the prongs redeployed to 5 cm. Another 2 mL was then injected in the same manner for 4 ml total.

### Gross pathology

Liver specimens that included the imageable gel were excised utilizing fluoroscopy to localize the gel. Tissues were flash-frozen in 2-mercaptoethanol that had been previously cooled in liquid nitrogen, and subsequently stored at – 80 °C before further processing. The tissue samples were sectioned along the needle shaft axis using a 3D-printed mold equipped with cutting slots at 5 mm intervals^63^. These sections were then photographed and examined for any signs of tissue fractures. The lengths of the major and minor axes were subsequently measured to evaluate the gel distribution.

### 2D Drug dose distribution across three needle devices

DOX distribution in tissue was determined after 4 mL X-ray imageable POL22 with 20 µg/mL of DOX was delivered with SEHN, MSHN, and MPIN-1 cm devices at 10 mL/h injection rates over 4 mL. In addition, with the same DOX dose, two MPIN techniques were performed as depicted in 2.7.7. The techniques were 2 and 5 without rotation at 100 mL/h with 4 mL and 2 mL respectively. The 5 mm tissue section (2.7.8) were imaged under an In vivo Imaging System (IVIS III, PerkinElmer, Whaltham, MA) (λ_ex_ = 480 nm and λ_em_ = 620 nm) and CBCT (80 kVp). To determine DOX distribution profiles, the fluorescence intensity of DOX was determined along a line through the center of the injection using the Living Image software (PerkinElmer, Whaltham, MA). Drug concentration was estimated with fluorescence intensity compared to a calibration curve based on 5, 10, and 20 µg/mL of DOX in a black 96-well plate (Greiner Bio-one, Monroe, NC). The maximum DOX concentration was not expected to exceed 20 µg/mL. The average of CBCT and optical imaging circularities, calculated from MATLAB were divided to determine similarity.

### Statistical analyses

Statistical analyses were performed using GraphPad Prism 9 (GraphPad Software, Boston, MA, www.graphpad.com). One-way ANOVA complemented by Tukey’s multiple comparison test was employed to compare gelation times, sphericities, and solidities. The *t* test was utilized to compare the sphericities and solidities of gel and non-gel specimens and to compare circularities of CBCT and optical images. Descriptive statistics were presented as mean ± standard deviation. Unless otherwise noted, all experiments were conducted in triplicate.

### Supplementary Information

Supplementary Information.

## Data Availability

The datasets used and/or analyzed during the current study available from the corresponding author on reasonable request.

## References

[1] Marabelle A (2018). Starting the fight in the tumor: Expert recommendations for the development of human intratumoral immunotherapy (HIT-IT). Ann. Oncol..

[2] Champiat S (2021). Intratumoral immunotherapy: From trial design to clinical practice. Clin. Cancer Res..

[3] Melero I, Castanon E, Alvarez M, Champiat S, Marabelle A (2021). Intratumoural administration and tumour tissue targeting of cancer immunotherapies. Nat. Rev. Clin. Oncol..

[4] Hassan R (2020). Phase 1 study of the immunotoxin LMB-100 in patients with mesothelioma and other solid tumors expressing mesothelin. Cancer.

[5] Weide B (2010). High response rate after intratumoral treatment with interleukin-2: Results from a phase 2 study in 51 patients with metastasized melanoma. Cancer.

[6] Andtbacka RH (2015). Talimogene laherparepvec improves durable response rate in patients with advanced melanoma. J. Clin. Oncol..

[7] Brody JD (2010). In situ vaccination with a TLR9 agonist induces systemic lymphoma regression: A phase I/II study. J. Clin. Oncol..

[8] Meric-Bernstam F (2022). Phase I dose-escalation trial of MIW815 (ADU-S100), an Intratumoral STING agonist, in patients with advanced/metastatic solid tumors or lymphomas. Clin. Cancer Res..

[9] Casares N (2005). Caspase-dependent immunogenicity of doxorubicin-induced tumor cell death. J. Exp. Med..

[10] Boiardi A (2005). Intratumoral delivery of mitoxantrone in association with 90-Y radioimmunotherapy (RIT) in recurrent glioblastoma. J. Neurooncol..

[11] Sun FF (2019). Oxaliplatin induces immunogenic cells death and enhances therapeutic efficacy of checkpoint inhibitor in a model of murine lung carcinoma. J. Recept Sig. Transd..

[12] Munoz NM (2021). Influence of injection technique, drug formulation and tumor microenvironment on intratumoral immunotherapy delivery and efficacy. J. Immunother. Cancer.

[13] Correa S (2021). Translational applications of hydrogels. Chem. Rev..

[14] Amende MT, Hariharan D, Peppas NA (1995). Factors influencing drug and protein-transport and release from ionic hydrogels. React. Polym..

[15] Kim SW, Bae YH, Okano T (1992). Hydrogels—swelling, drug loading, and release. Pharm. Res.-Dordr..

[16] Bruschi ML (2015). Strategies to Modify the Drug Release from Pharmaceutical Systems.

[17] Norouzi M, Nazari B, Miller DW (2016). Injectable hydrogel-based drug delivery systems for local cancer therapy. Drug Discov. Today.

[18] Nguyen QV, Huynh DP, Park JH, Lee DS (2015). Injectable polymeric hydrogels for the delivery of therapeutic agents: A review. Eur. Polymer J..

[19] Fan DY, Tian Y, Liu ZJ (2019). Injectable hydrogels for localized cancer therapy. Front. Chem..

[20] Chung CK (2020). Thermosensitive hydrogels as sustained drug delivery system for CTLA-4 checkpoint blocking antibodies. J. Control Release.

[21] Liu YF (2022). pH-sensitive peptide hydrogels as a combination drug delivery system for cancer treatment. Pharmaceutics.

[22] Chang GR (2019). Effective photodynamic therapy of polymer hydrogel on tumor cells prepared using methylene blue sensitized mesoporous titania nanocrystal. Mat. Sci. Eng. C-Mater..

[23] Fathi M (2019). Dual thermo-and pH-sensitive injectable hydrogels of chitosan/(poly(-isopropylacrylamide–itaconic acid)) for doxorubicin delivery in breast cancer. Int. J. Biol. Macromol..

[24] Alexandridis P, Holzwarth JF, Hatton TA (1994). Micellization of poly (ethylene oxide)-poly (propylene oxide)-poly (ethylene oxide) triblock copolymers in aqueous solutions: Thermodynamics of copolymer association. Macromolecules.

[25] Food and Drug Administration, Center for Drug Evaluation and Research. Inactive Ingredient Search for Approved Drug Products. Date accessed: April 21 (2022). https://www.accessdata.fda.gov/scripts/cder/iig/index.cfm?event=browseByLetter.page&Letter=P.

[26] Vadnere M, Amidon G, Lindenbaum S, Haslam JL (1984). Thermodynamic studies on the gel-sol transition of some pluronic polyols. Int. J. Pharmaceut..

[27] Huang L (2016). Thermo-sensitive composite hydrogels based on poloxamer 407 and alginate and their therapeutic effect in embolization in rabbit VX2 liver tumors. Oncotarget.

[28] Sheth RA (2020). Assessment of image-guided intratumoral delivery of immunotherapeutics in patients with cancer. Jama Netw. Open.

[29] Sudheendra D (2007). Comparison of three different needles for percutaneous injections. Cardiovasc. Int. Rad..

[30] Goel A. F. J., Murphy A, *et al*. Kilovoltage peak. Reference article. *Radiopaedia.org* (2021).

[31] Yan CQ (2010). Injectable solid hydrogel: Mechanism of shear-thinning and immediate recovery of injectable beta-hairpin peptide hydrogels. Soft Matter.

[32] Yan CQ, Schneider JP, Pochan DJ (2011). Injectable solid hydrogels as cell carriers: Mechanism of b-hairpin hydrogel shear thinning/immediate recovery and effects on cell payload. Abstr. Pap. Am. Chem. S.

[33] Chung CK (2020). Doxorubicin loaded poloxamer thermosensitive hydrogels: Chemical pharmacological and biological evaluation. Molecules.

[34] Marabelle A, Tselikas L, De Baere T, Houot R (2017). Intratumoral immunotherapy: using the tumor as the remedy. Ann. Oncol..

[35] Mandal A, Clegg JR, Anselmo AC, Mitragotri S (2020). Hydrogels in the clinic. Bioeng. Transl. Med..

[36] Hsu XL, Wu LC, Hsieh JY, Huang YY (2021). Nanoparticle-hydrogel composite drug delivery system for potential ocular applications. Polymers.

[37] Chatterjee S, Hui PCL, Kan CW, Wang WY (2019). Dual-responsive (pH/temperature) Pluronic F-127 hydrogel drug delivery system for textile-based transdermal therapy. Sci. Rep..

[38] Gao ZG, Lee DH, Kim DI, Bae YH (2005). Doxorubicin loaded pH-sensitive micelle targeting acidic extracellular pH of human ovarian A2780 tumor in mice. J. Drug Target.

[39] Gonzalez YM, Ghaly ES (2010). Modified drug release of poloxamer matrix by including water-soluble and water-insoluble polymer. Drug Dev. Ind. Pharm..

[40] Swietach P, Hulikova A, Patiar S, Vaughan-Jones RD, Harris AL (2012). Importance of intracellular pH in determining the uptake and efficacy of the weakly basic chemotherapeutic drug, doxorubicin. Plos One.

[41] Pec EA, Wout ZG, Johnston TP (1992). Biological-Activity of Urease Formulated in Poloxamer 407 after Intraperitoneal Injection in the Rat. J. Pharmaceut. Sci..

[42] Amalou H, Wood BJ (2013). Intratumoral gene therapy injections with a multipronged, multi-side hole needle for rectal carcinoma. Cardiovasc. Int. Rad.

[43] Haaga JR, Alfidi RJ (1976). Precise biopsy localization by computer tomography. Radiology.

[44] Solomon SB, Silverman SG (2010). Imaging in interventional oncology. Radiology.

[45] Ahmed M, Technology Assessment Committee of the Society of Interventional, R (2014). Image-guided tumor ablation: Standardization of terminology and reporting criteria–a 10-year update: Supplement to the consensus document. J. Vasc. Interv. Radiol..

[46] Dietrich CF (2020). Guidelines and good clinical practice recommendations for contrast-enhanced ultrasound (CEUS) in the Liver-Update 2020 WFUMB in Cooperation with EFSUMB, AFSUMB, AIUM, and FLAUS. Ultrasound Med. Biol..

[47] Abi-Jaoudeh N (2012). Multimodality image fusion-guided procedures: Technique, accuracy, and applications. Cardiovasc. Int. Rad..

[48] James AP, Dasarathy BV (2014). Medical image fusion: A survey of the state of the art. Inform. Fusion.

[49] Moore CM, Van Thiel DH (2013). Cirrhotic ascites review: Pathophysiology, diagnosis and management. World J. Hepatol..

[50] Jain RK (2014). Antiangiogenesis strategies revisited: From starving tumors to alleviating hypoxia. Cancer Cell.

[51] Ning S (1996). Intratumoral radioimmunotherapy of a human colon cancer xenograft using a sustained-release gel. Radiother. Oncol..

[52] Marabelle A, Kohrt H, Caux C, Levy R (2014). Intratumoral immunization: A new paradigm for cancer therapy. Clin. Cancer Res..

[53] Rajagopal JR (2021). Comparison of low dose performance of photon-counting and energy integrating CT. Acad. Radiol..

[54] Mikhail A (2017). Drug dose mapping using radiopaque, drug-eluting embolic beads following DEBTACE in rabbit VX2 using MDCT and micro CT. J. Vasc. Intervent. Radiol..

[55] Dickerscheid D, Lavalaye J, Romijn L, Habraken J (2013). Contrast-noise-ratio (CNR) analysis and optimisation of breast-specific gamma imaging (BSGI) acquisition protocols. EJNMMI Res..

[56] Rajagopal, J. R. *et al.* In *Medical Imaging 2021: Physics of Medical Imaging* 1187–1192 (SPIE, 2023).

[57] Raghavan, S. R. & Cipriano, B. H. In *Molecular Gels: Materials with Self-Assembled Fibrillar Networks* (eds. Richard, G. W. & Pierre, T.) 241–252 (Springer Netherlands, 2006).

[58] Baloglu E, Karavana SY, Senyigit ZA, Guneri T (2011). Rheological and mechanical properties of poloxamer mixtures as a mucoadhesive gel base. Pharmaceut. Dev. Technol..

[59] Edsman K, Carlfors J, Petersson R (1998). Rheological evaluation of poloxamer as an in situ gel for ophthalmic use. Eur. J. Pharmaceut. Sci..

[60] Russo J, Fiegel J, Brogden NK (2020). Rheological and drug delivery characteristics of poloxamer-based diclofenac sodium formulations for chronic wound site analgesia. Pharmaceutics.

[61] Hertig G (2017). Iodixanol as a contrast agent in a fibrin hydrogel for endodontic applications. Front. Physiol..

[62] Varble NA (2023). Morphometric characterization and temporal temperature measurements during hepatic microwave ablation in swine. PLoS One.

[63] Mikhail AS (2020). Liver-specific 3D sectioning molds for correlating in vivo CT and MRI with tumor histopathology in woodchucks (Marmota monax). PLoS One.

