# Supplementary material for: X-ray imageable, drug-loaded hydrogel that forms at body temperature for image-guided, needle-based locoregional drug delivery
Source: Sci Rep. 2024 Jun 10;14:13352. doi: 10.1038/s41598-024-64189-z (PMC11164888; doi:10.1038/s41598-024-64189-z)
Supplement: Supplementary file 1 — Supplementary Information. [file 41598_2024_64189_MOESM1_ESM.docx]

**Supplemental**

**Development and properties of an X-ray imageable drug-loaded POL formulation**

For POL17, POL18, and POL22 the gelation times were 41.3 ± 0.6 s, 27.8 ± 1.5 s, and 12 ± 1 s, respectively (Fig.1a) [(****) p<0.001; (***) p=0.0001;(**) p=0.0069]. The linear regression of gelation time as a function of POL concentration (%, w/v) was y= -5.238x + 124.7, r^2^=0.8. POL17 formulations with and without iodixanol did not form a gel (Fig.1b), therefore, gelation temperature was not determined. The gelation temperature for POL18 increased by 7% from 27.2 + 0.1 °C to 28.7 + 0.6 °C with the addition of iodixanol (p = 0.0045) (Fig.S1c and Fig.S1d). In the absence of iodixanol, the gelation temperature of POL22 was determined to be 22.2 + 0.3 °C, based on the G' and G" values shown in Fig. S1e. When iodixanol was added to POL22, the gelation temperature increased by 42% to 31.6 + 0.5 °C, compared to native POL22 (p < 0001) (Fig. 1f).

|  |
| --- |
| **Fig.S1.** (a) Gelation times of various POL concentrations ranging from 17 to 22% (w/v). (b) Temperature ramp from 5°C to 37°C imageable and non-imageable POL17, (c) non-imageable POL18, (d) imageable POL18, (e) non-imageable POL22, and (f) imageable POL22. n=3 for all experiments. |

All POL18 and POL 22 formulations formed hydrogels suitable for injection as demonstrated by their viscoelastic behavior. POL18 and POL22, with or without iodixanol, showed viscoelastic properties characteristic for gels as evidenced by G’ exceeding G” values (Fig.S2a, and Fig.S2b). Overall, the addition of iodixanol in the POL formulations resulted in softer hydrogels with lower G' in comparison with the POL alone. The G’ value of POL22 was 3.1 times higher than that of POL22 + iodine (p < 0.0001). The G’ of POL18 and POL18 + iodine did not change. As the percentage of POL increased, the complex viscosity and G’ values also increased; nevertheless, the inclusion of iodixanol in the formulation caused a decrease in G’.

|  |
| --- |
| **Fig.S2.** (a) G’, G”, and complex viscosity of both non-imageable and imageable POL18 and (b) non-imageable and imageable POL22. n=3 for all experiments. Error bars represent standard deviations of mean value. |

POL18 and POL22 with and without iodixanol displayed thixotropic behavior and recovered after a shearing event (Fig.S3a and, Fig.S3b). The gels were capable of recovering their initial G’ values after high strain. All the materials were capable of immediately recovering their gel state. POL22 and POL22 + iodixanol gels displayed a small decrease in their G’ after thinning (Fig.S3b).

Formulations of POL22 + Iodine with DOX displayed G' < G" during high strain and G' > G" immediately after high strain, indicating a liquid-like behavior during injection and expected gel formation immediately post-injection (Fig.S3c).

|  |
| --- |
| **Fig.S3.** Thixotropic properties and viscoelastic behavior of various poloxamer formulations. Storage modulus (G’) for (a) POL18, and (b) POL22 with and without iodine before, during, immediately after, 10 minutes after, and 56 minutes after strain. (c) Storage modulus (G’) for iodinated POL22 with 2mg/mL, and 5mg/mL of DOX, before, during, immediately after, 10 minutes after, and 56 minutes after strain. n=3 for all experiments. |

A summary of measured G’ values can be found in Table S1.

| **Table S1.** Storage modulus (G’) values of imageable and nonimageable gels, as well as gels with DOX incorporated into the imageable gel before 1000% shear stress, and immediately after, 10 minutes after, and 56 minutes after application of shear stress. + denotes standard deviations. | | | | |
| --- | --- | --- | --- | --- |
| **Sample** | **G’ before shear stress (Pa)** | **G’ right after**  **shear stress (Pa)** | **G’ 10 min after strain (Pa)** | **G’ 56 min after strain (Pa)** |
| POL18 | 9563.5  + 150.9 | 6868.7  + 275.9 | 8164.1  +263.5 | 10015  +240.7 |
| POL18 + Iodine | 8608.3  + 946.3 | 5898.9  +734.5 | 7191.9  +540.6 | 8337.5  +1036.5 |
| POL22 | 17163.5  + 644.2 | 12117.6  + 435.3 | 13336.5  +547.3 | 13990.8  +458.3 |
| POL22 + Iodine | 6848.7  + 365.2 | 3703.8  +1439.3 | 5966.2  +490.8 | 6489.9  +501.5 |
| POL22 + Iodine + DOX (2 mg/mL) | 13546.7  + 435.1 | 8846.4  + 681.2 | 9759.7  +684.2 | 10142.7  + 685.6 |
| POL22 + Iodine + DOX (5 mg/mL) | 16024.9  + 624.5 | 10392.5  + 840.4 | 11549.6  + 840.4 | 12093.8  + 756.1 |
| POL22 + Iodine + DOX (10 mg/mL) | 11006.8  +251.8 | 8475.5  + 542.3 | 8475.5  + 542.3 | 9979.1  + 546.4 |

The flow points for POL18 (Fig.S4a) and iodinated POL18 (Fig.S4b) were found to be 5.9 ± 1.4 and 4.1% ± 0.5 respectively. For POL22 (Fig.S4c) and iodinated POL22 (Fig.S4d), the flow points were 5.4% ± 0.00, and 4.1% ± 0.5. When 2, and 5mg/mL of DOX were incorporated into POL22 + iodixanol (Fig.S4e), the critical strain values recorded were 6.5% ± 0.8, and 10.9% ± 0.00, respectively. The frequency range tested showed gel behavior at 37 °C for POL18 and POL22, both with and without iodixanol, as well as POL22 containing both iodixanol and DOX (at 2, and 5 mg/mL). G’ exceeded G”, indicating that the material behaved as a viscoelastic gel and remained stable over time (Fig.S5a-S5f).

|  |
| --- |
| **Fig.S4.** (a) Oscillation strain sweep for POL18 formulations without iodine, and with (b) iodine. (c) Oscillation strain sweep for POL22 formulations without iodine, and with (d) iodine. (e) Oscillation strain sweep for iodinated POL22 formulations with 2, and 5mg/mL of DOX. n=3 for all experiments. |

|  |
| --- |
| **Fig.S5.** (a) Frequency sweep for POL18 formulations without iodine, and with (b) iodine. (c) Frequency sweep for POL22 formulations without iodine, and with (d) iodine. (e) Frequency sweep for iodinated POL22 formulations with 2, and 5mg/mL of DOX. n=3 for all experiments. |

|  |
| --- |
| **Fig.S6.** Zoom of elution profiles for 2, 5, and 10 mg/ml DOX from the gel matrix, alongside their respective control groups which consist of DOX alone with iodine. n=3 for all experiments. Error bars represent standard deviations of mean value. |

***Ex vivo* evaluation of injection parameters for three devices in bovine liver**

Determining the “critical volume” of injections (and threshold of extravasation) is of paramount importance as an efficiency, efficacy, and safety parameter. The SEHN technique at 1000 mL/h was utilized to evaluate injections of 4, 8.6, and 14 mL to ascertain the maximum volume that could be injected without causing extravasation. Fig.S6 illustrates the outcomes of three distinct segmented 14 mL injections of POL22 + Iodine into bovine livers. The green color-coded segmentation is a localized injection without evidence of extravasation, in contrast to the blue- and yellow-coded segmentations that spread into nearby vessels. Even though localized deposition of the gel was attainable across all tested volumes, the likelihood of extravasation increased with increases in volume. Extravasation is undesirable due to its potential for vascular spread of the gel, leading to nontarget delivery, which could amplify adverse effects and undermine therapeutic efficacy^5,6^. Hence, for further evaluations of different needle devices, 4 mL injections were chosen due to their lower degree of extravasation. The imaging parameters obtained from these injections are presented in Table S3.

The sphericities of the infused material were 0.9 ± 0.3, 0.8 ± 0, and 0.8 ± 0 for 4, 8.6, and 14 mL respectively. The solidities of the materials were 0.9 ± 0.4, 0.9 ± 0, and 0.8 ± 0 for 4, 8.6, and 14 mL respectively. The sphericity and solidity of the deposited gel decreased as the volume increased due to a higher degree of extravasation although these values were not statistically significant.


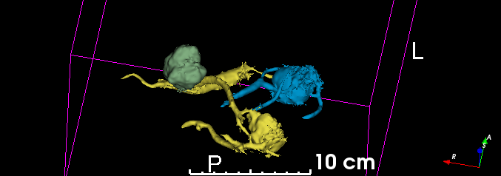


**Fig.S7.** 3D reconstruction of 14 mL of POL22 injected into ex vivo bovine liver, distinguishing between localized (green) and non-localized (blue and yellow) depositions at an infusion rate of 1000 mL/h.

| **Table S2.** Parameters for 4, 8.6, and 14 mL infused gels: infused volume, mean radiopacity, experimental volume, and percent volume error. HU = Hounsfield Units. | | | |
| --- | --- | --- | --- |
| **Infused volume (mL); Injection rate (mL/h)** | **Mean radiopacity (HU)** | **Computed volume from segmentation (mL)** | **% Error of volume** |
| 4; 1000 | 858.6 + 82.8 | 5.0 + 0.5 | 19.7 |
| 8.6; 1000 | 696.4 + 38.8 | 10 + 2.2 | 14.5 |
| 14; 1000 | 621.16 + 430.6 | 17.5 + 1.7 | 20.0 |

**Fig.S8.** Morphometric analysis of POL22 injections. Sphericity and solidity as a function of the injected volume using the SEHN needle device at an infusion rate of 1000 mL/h. n=3 for all experiments. Error bars represent standard deviations of mean value.

| **Table S3.** Sphericities and solidities for different needle devices. MPIN (2cm) produced three different depositions from each tip (A, B, and C), therefore, solidities and sphericities are reported for each deposition. | | | | | | | | | | | |
| --- | --- | --- | --- | --- | --- | --- | --- | --- | --- | --- | --- |
| **Needle device** | **SEHN** | | | | **MSHN** | | | | **MPIN (1cm)** | | |
| **Volume** | **Sphericity (a.u)** | | | **Solidity (a.u)** | **Sphericity (a.u)** | | | **Solidity (a.u)** | **Sphericity (a.u)** | | **Solidity (a.u)** |
| 1mL | 0.9 +0.0 | | | 0.8 +0.1 | 0.8 +0.0 | | | 0.8+0.0 | 0.6+0.0 | | 0.4+0.3 |
| 2mL | 0.9 +0.1 | | | 1 +0.3 | 0.8 +0.1 | | | 0.8+0.0 | 0.6+0.0 | | 0.7+0.1 |
| 3mL | 0.9 +0.0 | | | 0.9 +0.0 | 0.8 +0.0 | | | 0.9+0.0 | 0.7+0.1 | | 0.8+0.0 |
| 4mL | 0.9 +0.1 | | | 0.9 +0.1 | 0.8 +0.0 | | | 0.9+0.0 | 0.7+0.0 | | 0.8+0.0 |
| **Needle device** | | **MPIN (2cm)** | | | | | | | | | |
|  | | **Sphericity (a.u)** | | | | | **Solidity (a.u)** | | | | |
| **Volume** | | Tip A | Tip B | | | Tip C | Tip A | | | Tip B | Tip C |
| 1mL | | 0.9+0.0 | 0.9+0.1 | | | 0.9+0.1 | 0.9+0.0 | | | 0.8+0.1 | 0.6+0.5 |
| 2mL | | 0.9+0.0 | 0.8+0.1 | | | 0.8+0.1 | 0.9+0.0 | | | 0.8+0.1 | 0.9+0.1 |
| 3mL | | 0.9+0.0 | 0.8+0.1 | | | 0.8+0.1 | 0.9+0.0 | | | 0.8+0.2 | 0.9+0.1 |
| 4mL | | 0.9+0.0 | 0.8+0.1 | | | 0.8+0.1 | 0.9+0.0 | | | 0.8+0.2 | 0.9+0.1 |

| **Table S4.** Circularities and solidities for SEHN and MSHN needle devices. | | | | | | |
| --- | --- | --- | --- | --- | --- | --- |
| **Needle device** | **SEHN** | | **MSHN -needle plane-** | | **MSHN -perpendicular to needle-** | |
| **Volume** | **Circularity (a.u)** | **Solidity (a.u)** | **Circularity**  **(a.u)** | **Solidity (a.u)** | **Circularity**  **(a.u)** | **Solidity (a.u)** |
| 1mL | 0.8+0.1 | 0.9+0.0 | 0.7+0.1 | 0.9+0.0 | 0.7+0.0 | 0.9+0.0 |
| 2mL | 0.8+0.1 | 1.0+0.0 | 0.7+0.1 | 0.9+0.0 | 0.7+0.1 | 0.9+0.0 |
| 3mL | 0.8+0.1 | 0.9+0.0 | 0.8+0.1 | 0.9+0.0 | 0.8+0.1 | 0.9+0.0 |
| 4mL | 0.8+0.1 | 0.9+0.0 | 0.8+0.1 | 0.9+0.0 | 0.7+0.1 | 0.9+0.0 |

| **Table S5.** Overview of resultant MPIN morphologies dependent on injection techniques | |
| --- | --- |
| **Technique** | **Description** |
| 1 | MPIN deployed with a short tip of 2 cm, infusing 2 mL, retracting to 1 cm, and infusing an additional 2 mL, without rotation. This method resulted in three elliptical gel collections with a major axis of 16.2 ± 2 mm and a minor axis of 9.2 ± 1.3 mm. |
| 2 | Short tip of MPIN was deployed 2 cm and 4 mL gel was infused, without rotation. This yielded a conical shape with a base diameter of 2.6 ± 2 mm and a height of 22.9 ± 2 mm. |
| 3 | MPIN deployed 5 cm and 4 mL of gel infused while retracting the needle in 1 cm increments, without rotation, forming three chains of interconnected gel collections with a diameter of 7 ± 0.4 mm at the base of the pyramidal shape. |
| 4 | Infused 4 mL of gel as in technique 3, rotated the needle 60 degrees, and repeated the injection resulting in a star shape made of gel collections measuring 7.7 ± 0.6 mm interconnected at the base of the pyramidal shape. A total of 8 mL was injected. |
| 5 | MPIN deployed 5 cm and 2 mL of gel injected while retracting the needle tips in 1 cm increments, rotated the needle 60 degrees, and repeated the injection process for a total of 4 mL, yielding a similar structure of interconnected gel collections measuring 6 ± 0.2 mm at the base of the pyramidal shape. |

**References**

1 Chung, C. K. *et al.* Doxorubicin Loaded Poloxamer Thermosensitive Hydrogels: Chemical, Pharmacological and Biological Evaluation. *Molecules* **25** (2020). <https://doi.org:10.3390/molecules25092219>

2 Marabelle, A., Tselikas, L., De Baere, T. & Houot, R. Intratumoral immunotherapy: using the tumor as the remedy. *Annals of Oncology* **28**, xii33-xii43 (2017).

3 Mandal, A., Clegg, J. R., Anselmo, A. C. & Mitragotri, S. Hydrogels in the clinic. *Bioeng Transl Med* **5** (2020). <https://doi.org:ARTN> e10158

10.1002/btm2.10158

4 Goel A, F. J., Murphy A, et al. Kilovoltage peak. Reference article. *Radiopaedia.org* (2021).

5 Munoz, N. M. *et al.* Influence of injection technique, drug formulation and tumor microenvironment on intratumoral immunotherapy delivery and efficacy. *J Immunother Cancer* **9** (2021). <https://doi.org:ARTN> e001800

10.1136/jitc-2020-001800

6 Sheth, R. A. *et al.* Assessment of Image-Guided Intratumoral Delivery of Immunotherapeutics in Patients With Cancer. *Jama Netw Open* **3** (2020). <https://doi.org:ARTN> e207911

10.1001/jamanetworkopen.2020.7911
